# Mechanoinflammation in osteoarthritis: knowledge evolution, cellular interactions, and translational research priorities

**DOI:** 10.3389/fimmu.2026.1955650

**Published:** 2026-10-09

**Authors:** Bo Wang, Yuge Li, Jiaxing Huang, Yin Lian, Lina Gao, Suya Zhang, Sijin Tang, Dandan Li, Renxuan Yang, Huai Wu

**Affiliations:** 1 Shenzhen Pingle Orthopedic Hospital (Shenzhen Pingshan Traditional Chinese Medicine Hospital), Shenzhen, China; 2 Shenzhen Orthopedic Hospital Affiliated to Guangzhou University of Chinese Medicine, Shenzhen, China; 3 The Seventh Affiliated Hospital, Sun Yat-sen University, Shenzhen, China; 4 Department of Clinical Medical Research Center, The Second Clinical Medical College of Jinan University (Shenzhen People’s Hospital), Shenzhen, China

**Keywords:** inflammation, mechanobiology, mechanoinflammation, mechanotransduction, osteoarthritis, synovitis, tissue crosstalk

## Abstract

**Background:**

Osteoarthritis (OA) is increasingly recognized as a whole-joint disorder in which mechanical stress, cellular mechanosensing, and inflammatory responses interact across joint tissues. Although OA mechanoinflammation has attracted growing attention, its literature remains distributed across mechanobiology, inflammation research, molecular medicine, and translational research. This study aimed to map the knowledge structure and temporal development of OA mechanoinflammation and to characterize relationships among mechanosensing, inflammatory responses, tissue crosstalk, and emerging research priorities.

**Methods:**

Publications related to OA mechanoinflammation were retrieved from the Web of Science Core Collection and PubMed from January 1, 2000 to May 23, 2026. Bibliometric and knowledge-map analyses characterized publication trends, collaboration patterns, reference co-citation structures, author keyword themes, journal-level knowledge flows, and sensitivity to database source, retrieval dependence, and clustering parameters. Keyword–reference correspondence was evaluated using year-stratified permutation testing and bootstrap confidence intervals, and temporal use of reference clusters was assessed using an annual citation-opportunity-adjusted analysis.

**Results:**

Research output increased markedly in recent years. Under the primary analytical configuration, reference co-citation and author keyword analyses each yielded four interpretable intermediate-resolution domains. Validated keyword–reference correspondence identified selective relationships linking mechanotransduction-related themes with the mechanical-loading and mechanosensitive-channel knowledge bases, together with a significant relationship between the broader inflammatory-degeneration theme and contemporary OA pathogenesis, aging, and tissue crosstalk. After adjustment for citation opportunity, recent years showed greater relative use of the contemporary OA/tissue-crosstalk and mechanosensitive-channel reference domains, with substantial year-to-year heterogeneity. Sensitivity analyses showed high stability of the principal reference-based structures and identified greater variability at keyword-domain boundaries.

**Conclusions:**

OA mechanoinflammation is an expanding interdisciplinary research area linking mechanical context, cellular mechanosensing, inflammatory responses, and whole-joint remodeling at the literature level. The mapped structures and recent temporal patterns identify testable priorities for multimodal phenotyping, biomarker validation, mechanistically relevant models, and combined mechanical-inflammatory intervention research. These translational directions remain hypothesis-generating and require prospective experimental and clinical validation.

## 1 Introduction

Osteoarthritis (OA) is a highly prevalent degenerative joint disease and a major cause of chronic pain, functional limitation, and disability worldwide (1). Although OA was traditionally viewed mainly as a disease of articular cartilage wear and structural degeneration, it is now increasingly recognized as a whole-joint disorder involving cartilage, subchondral bone, synovium, meniscus, infrapatellar fat pad, and periarticular tissues (2, 3). Within this broader disease framework, inflammation is regarded as an important contributor to OA onset and progression. Synovitis, macrophage activation, inflammatory cytokines, chemokines, catabolic enzymes, and pain-related inflammatory mediators can shape the local joint microenvironment and contribute to cartilage degeneration and tissue remodeling (4–6). Within this whole-joint context, the term mechanoinflammation describes the interaction between mechanical disturbance, cellular mechanosensing, inflammatory activation, and tissue remodeling in OA. In this usage, the inflammatory component encompasses responses of both resident joint cells and immune populations rather than denoting a discrete immune-cell-centered OA subtype.

Mechanical factors are central to OA pathogenesis. Abnormal mechanical loading, joint instability, excessive compression, altered shear stress, traumatic injury, obesity-related biomechanical burden, and changes in extracellular matrix stiffness can all affect joint tissue homeostasis (7). Joint-resident cells, including chondrocytes, synoviocytes, and osteoblast-lineage cells, can sense and respond to these mechanical cues through mechanotransduction pathways. These cells are not only structural components of the joint but can also act as inflammatory responder cells, producing cytokines, chemokines, catabolic enzymes, and other mediators when exposed to pathological mechanical conditions. Mechanosensitive ion channels, primary cilia, integrin-related signaling, cytoskeletal remodeling, and matrix-associated signaling cascades have been implicated in the conversion of physical stimuli into biological responses (8–11). Under abnormal or sustained mechanical stress, mechanotransduction responses may promote catabolic activity, inflammatory signaling, matrix degradation, and tissue damage (12–14).

The interaction between mechanobiology and inflammation has therefore become an important research direction in OA. Mechanical stress can regulate inflammatory responses by influencing cytokine production (9), macrophage-related signaling (15), synovial activation (16), inflammasome-related pathways (15, 17), and mechanosensitive channels such as Piezo1 and TRPV4 (18, 19). This response involves interactions among immune cells and joint-resident stromal cells. Synovial macrophages may amplify mechanically triggered inflammation, whereas chondrocytes, synovial fibroblasts, osteoblast-lineage cells, and mesenchymal stromal cells can participate in cytokine production, inflammasome signaling, metabolic reprogramming, and matrix-degrading responses. Inflammatory mediators may also alter matrix composition and stiffness (20, 21), sensitize cellular mechanotransduction (22), and reduce the capacity of joint tissues to maintain mechanical homeostasis. These changes may create a feed-forward process in which inflammation modifies the mechanical environment and cellular mechanosensitivity, thereby increasing subsequent inflammatory and catabolic responses to mechanical stimulation (9, 23). This reciprocal relationship provides a conceptual bridge between biomechanical injury, inflammatory activation, and progressive joint degeneration, and places OA within a broader framework involving stromal-cell responses and crosstalk among cartilage, synovium, subchondral bone, and other joint tissues (24, 25).

Despite growing interest in this field, the literature on mechanobiology-related inflammation in OA remains dispersed across orthopedics, rheumatology, immunology, molecular biology, biomechanics, materials science, and translational research. Individual studies have examined specific mechanisms, such as chondrocyte mechanotransduction (26, 27), synovial inflammation (10), mechanosensitive channels (28, 29), cytokine-mediated matrix remodeling (11), post-traumatic OA (30), or obesity-related biomechanical stress (31). These studies have provided important mechanistic insights, but mechanical loading, inflammatory signaling, cellular responses, and tissue remodeling are often examined within partly separate experimental or disciplinary contexts. The literature-level organization linking mechanical exposure, cellular mechanosensing, inflammatory responses, stromal and immune-cell interactions, and whole-joint remodeling has not been systematically characterized.

Bibliometric analysis provides quantitative tools for mapping scientific production, collaboration patterns, citation structures, and thematic evolution (32). When combined with knowledge-map visualization, it can identify influential intellectual bases, emerging topics, and cross-disciplinary connections that may not be evident from narrative review alone. This approach is particularly useful for OA mechanobiology and inflammation because the field spans multiple tissue compartments, cell types, molecular mechanisms, disease contexts, and clinical translation pathways (33).

In this study, we conducted a dual-database bibliometric and knowledge-map analysis of mechanobiology-related inflammation in OA from 2000 to 2026. Using publications retrieved from the Web of Science Core Collection and PubMed, we examined the developmental trajectory, intellectual bases, active thematic domains, structural correspondence between keyword and co-citation clusters, temporal changes in knowledge prominence, and cross-disciplinary research patterns. Particular attention was given to the literature-level relationships among mechanical exposure, cellular mechanosensing, inflammatory signaling, stromal and immune-cell responses, and whole-joint tissue remodeling. We further developed a literature-informed framework to organize these findings and to identify hypothesis-generating priorities for phenotyping, biomarker validation, mechanistically relevant models, target evaluation, and future stratified intervention studies.

## 2 Methods

### 2.1 Data sources and search strategy

Publications on mechanobiology-related inflammation in osteoarthritis (OA) were retrieved from the Web of Science Core Collection (WoSCC) and PubMed. The search covered publications published between January 1, 2000 and May 23, 2026.

The search strategy combined terms related to OA, mechanobiology, and inflammation. In WoSCC, the search was performed in the Topic field (TS), which covers titles, abstracts, author keywords, and Keywords Plus. The WoSCC query was as follows:

TS=((osteoarthritis OR osteoarthritic OR “knee osteoarthritis” OR KOA OR “degenerative joint disease”) AND (mechanobiology OR mechanotransduction OR “mechanical loading” OR “mechanical stress” OR “mechanical stimulation” OR “mechanical signal*” OR “mechanical force*” OR “mechanical strain” OR mechanosensitive OR “mechanically activated” OR “mechanosensitive channel*” OR “mechanosensitive ion channel*” OR “mechanically activated channel*” OR “stretch-activated channel*” OR PIEZO1 OR PIEZO2 OR “Piezo channel*” OR TRPV4 OR “transient receptor potential vanilloid 4”) AND (inflammation OR inflammatory OR “inflammatory response” OR synovitis OR synovium OR synovial OR macrophage* OR monocyte* OR cytokine* OR chemokine*)).

Using this strategy, WoSCC initially returned 1,775 records. After restricting the document types to articles and reviews, language to English, and publication year to 2000 onward, 740 records were retained.

In PubMed, the search combined Medical Subject Headings (MeSH) and free-text terms in titles and abstracts. The query was as follows:

(“Osteoarthritis”[Mesh] OR osteoarthritis[Title/Abstract] OR osteoarthritic[Title/Abstract] OR “knee osteoarthritis”[Title/Abstract] OR KOA[Title/Abstract] OR “degenerative joint disease ”[Title/Abstract]) AND (mechanobiology[Title/Abstract] OR mechanotransduction[Title/Abstract] OR “mechanical loading”[Title/Abstract] OR “mechanical stress”[Title/Abstract] OR “mechanical stimulation”[Title/Abstract] OR “mechanical signal”[Title/Abstract] OR “mechanical signals”[Title/Abstract] OR “mechanical force”[Title/Abstract] OR “mechanical forces” [Title/Abstract] OR “mechanical strain”[Title/Abstract] OR mechanosensitive[Title/Abstract] OR “mechanically activated”[Title/Abstract] OR “mechanosensitive channel”[Title/Abstract] OR “mechanosensitive channels”[Title/Abstract] OR “mechanosensitive ion channel”[Title/Abstract] OR “mechanosensitive ion channels”[Title/Abstract] OR “mechanically activated channel”[Title/Abstract] OR “mechanically activated channels”[Title/Abstract] OR “stretch-activated channel”[Title/Abstract] OR “stretch-activated channels”[Title/Abstract] OR PIEZO1[Title/Abstract] OR PIEZO2[Title/Abstract] OR “Piezo channel”[Title/Abstract] OR “Piezo channels”[Title/Abstract] OR TRPV4[Title/Abstract] OR “transient receptor potential vanilloid 4”[Title/Abstract]) AND (inflammation[Title/Abstract] OR inflammatory[Title/Abstract] OR “inflammatory response”[Title/Abstract] OR synovitis[Title/Abstract] OR synovium[Title/Abstract] OR synovial[Title/Abstract] OR macrophage[Title/Abstract] OR macrophages[Title/Abstract] OR monocyte[Title/Abstract] OR monocytes[Title/Abstract] OR cytokine[Title/Abstract] OR cytokines[Title/Abstract] OR chemokine[Title/Abstract] OR chemokines[Title/Abstract]).

The mechanobiology component of the search strategy was designed primarily around process-level descriptors rather than an exhaustive enumeration of individual mechanical stimuli, cellular structures, or mechanosensors. Broad terms such as mechanobiology, mechanotransduction, mechanical loading, mechanical stress, mechanical stimulation, mechanical force, mechanical strain, and mechanosensitive/mechanically activated were intended to capture studies addressing diverse mechanobiological processes. PIEZO1/2 and TRPV4 were included as high-specificity supplementary terms to reduce the possibility of missing studies indexed primarily by specific mechanosensitive-channel names. Accordingly, mechanobiological examples considered during eligibility assessment, such as compression, hydrostatic pressure, joint instability, primary cilia, and integrin-related signaling, were not intended to correspond one-to-one with individual retrieval terms.

PubMed initially returned 578 English-language records. After restricting the publication year to 2000 onward, 568 records were retained.

WoSCC served as the primary bibliometric data source because it provides relatively complete and standardized bibliographic metadata, including citation information and journal-based subject categories. PubMed was included mainly to improve biomedical topic coverage. Because PubMed records are not directly equivalent to WoSCC records in terms of citation structure and bibliometric fields, WoSCC records were preferentially retained for analyses involving citation relationships, source journals, and subject categories, whereas PubMed was used to identify additional biomedically relevant records that were not captured by WoSCC and to improve the completeness of topic coverage.

### 2.2 Literature screening and data collection

Records retrieved from WoSCC and PubMed were exported in compatible bibliographic formats and processed in the working directory. The two datasets were merged, and duplicate records were identified using DOI matching, PMID matching, title comparison, and manual verification when necessary. When duplicate records were identified across databases, the WoSCC record was retained as the primary bibliographic record. After deduplication, 508 duplicate records were removed, and 800 unique records remained for eligibility screening.

Eligibility was determined according to predefined inclusion and exclusion criteria. Records were considered eligible if they focused on OA or OA-relevant joint tissue degeneration, including knee OA, post-traumatic OA, osteoarthritic cartilage, synovium, or subchondral bone; addressed mechanobiology-related processes, such as mechanical loading, mechanical stress, mechanical strain, compression, hydrostatic pressure, joint instability, exercise-related mechanical stimulation, mechanotransduction, mechanosensitive or mechanically activated channels, Piezo1/Piezo2, TRPV4, primary cilia, or comparable mechanobiological pathways; and investigated inflammatory mechanisms, including inflammatory responses, synovitis, macrophage or monocyte involvement, cytokines, chemokines, inflammatory pain, synovial fibroblast activation, or inflammation-related signaling in OA-relevant tissues.

Records were excluded if they did not focus on OA or OA-relevant joint degeneration; focused on general biomechanics, gait, prosthesis, arthroplasty, spinal disorders, finite element modeling, or mechanical engineering topics without OA-relevant inflammatory mechanisms; focused on OA inflammation, biomarkers, drug delivery, metabolism, or general pathophysiology without mechanobiology-related content; or were editorials, corrigenda, book chapters, procedural chapters, case reports, conference abstracts, letters, or other non-research items outside the planned article and review scope.

The 800 deduplicated records were independently reviewed by two investigators based on titles, abstracts, keywords, and bibliographic information. Full texts or publisher records were consulted when bibliographic information was insufficient to determine eligibility. Disagreements were resolved through discussion and, when required, adjudication by a third reviewer. After screening, 554 publications were finally included in the bibliometric analysis.

Because this study was designed as a bibliometric analysis rather than a conventional systematic review, the literature identification and screening process was documented using a PRISMA 2020-style flow diagram to improve reporting transparency, rather than as a formal PRISMA-compliant evidence-synthesis workflow (**Figure 1**).

**Figure 1:**
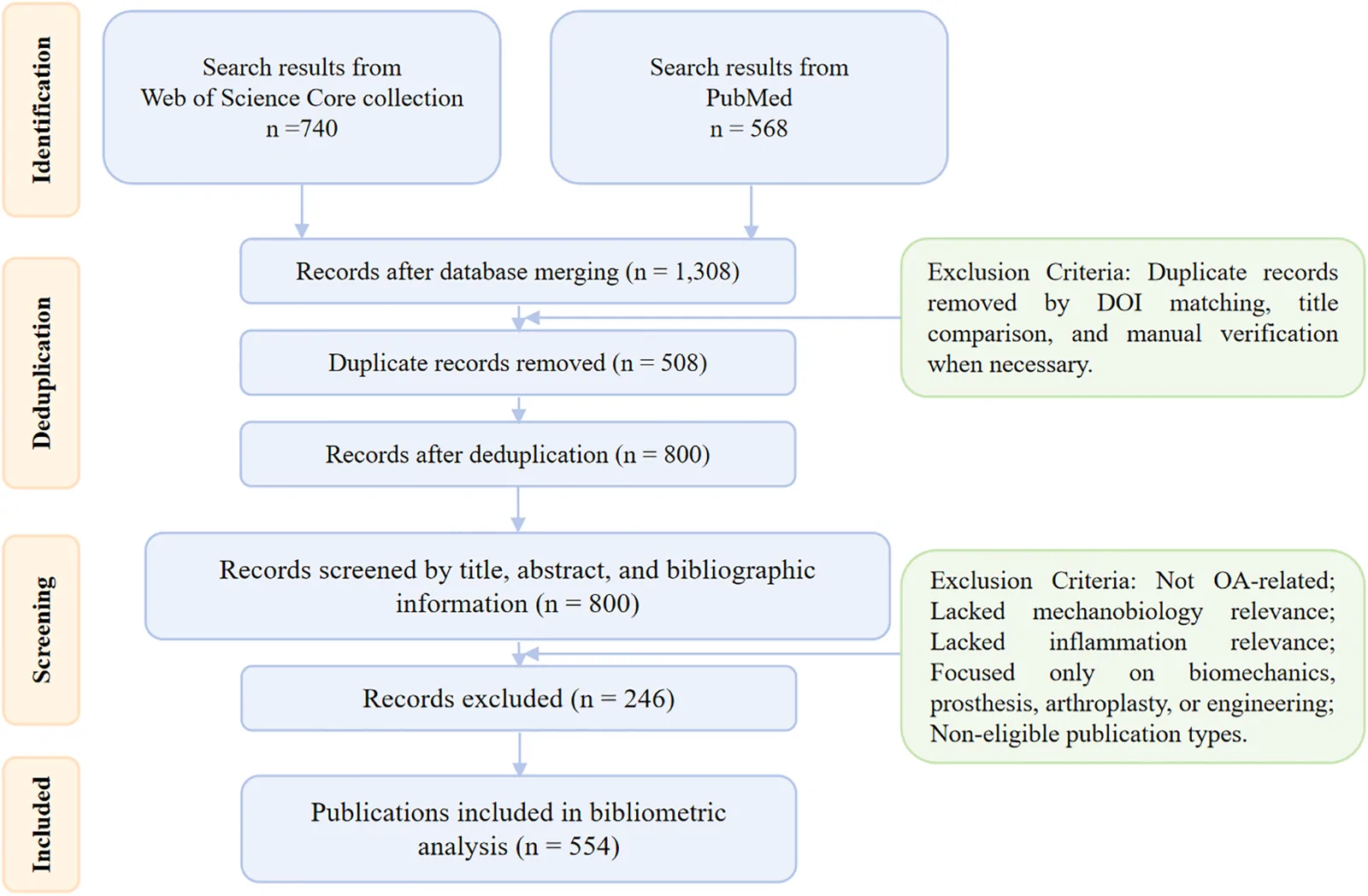
PRISMA-style flow diagram of literature identification and screening. Publications were retrieved from the Web of Science Core Collection and PubMed on May 23, 2026. After database-specific restrictions, 740 records from the Web of Science Core Collection and 568 records from PubMed were retained and merged. After removing 508 duplicate records, 800 records were screened by title, abstract, and bibliographic information. A total of 246 records were excluded according to the predefined eligibility criteria, and 554 publications were finally included in the bibliometric analysis. This PRISMA-style flow diagram was used to document the literature identification and screening process for reporting transparency.

### 2.3 Bibliometric analysis

Bibliometric analysis was performed using R (version 4.5.1) with the bibliometrix package, and network visualization was conducted using VOSviewer (version 1.6.20). The analysis included annual publication trends, contribution analysis of countries, institutions, authors, and journals, collaboration network analysis, author keyword co-occurrence analysis, reference co-citation analysis, cited-source co-citation analysis, journal bibliographic coupling analysis, and dual-map overlay analysis of journals. For VOSviewer-based analyses, full counting was used. The minimum threshold was set at three publications for country/region, institution, and author collaboration analyses, ten citations for reference co-citation analysis, twenty citations for cited-source co-citation analysis, five occurrences for author keyword co-occurrence analysis, and three documents for journal bibliographic coupling analysis. For VOSviewer clustering, the resolution was set to 1, the minimum cluster size was set to 3, and the option to merge small clusters was selected. These thresholds and clustering settings were selected to retain the major structure of the field while avoiding overly sparse networks.

In VOSviewer-based network maps, nodes represent countries, institutions, authors, references, keywords, or journals depending on the analytical unit. Node size reflects publication output, occurrence frequency, citation frequency, or total link strength, as appropriate. Links indicate collaborative, co-occurrence, co-citation, or bibliographic coupling relationships, and thicker links represent stronger relationships. Clusters were interpreted according to representative nodes, high-frequency terms, high-link-strength items, and the content of key publications within each cluster.

Before analysis, bibliographic metadata were checked and standardized. Author names, institutional names, country or region names, journal titles, and keywords were reviewed to reduce inconsistencies caused by spelling variation, abbreviations, singular and plural forms, and synonymous expressions. Keyword cleaning was performed conservatively to preserve the original meaning of the records while improving the consistency of term-based analyses. After cross-database deduplication and eligibility screening, 542 of the 554 included publications were represented by native WoSCC records, whereas 12 were PubMed-only records. For publications indexed in both databases, the WoSCC record was preferentially retained. The 12 PubMed-only records were linked to corresponding OpenAlex works using exact DOI matching to harmonize bibliographic metadata and obtain cited-reference information. All 12 records were uniquely matched. PMID, normalized title, and publication year were cross-checked against the original PubMed export and were concordant for all 12 records, with no ambiguous or unmatched records identified (**Supplementary Table S6**). OpenAlex metadata were accessed on May 24, 2026. For the PubMed-only records, OpenAlex referenced_works were converted to standardized cited-reference fields for compatibility with the merged bibliographic dataset. OpenAlex citation counts were not used as substitutes for WoSCC citation counts. Thus, cited-reference information in the merged dataset originated from native WoSCC records for 542 publications and from OpenAlex-linked reference lists for the 12 PubMed-only publications. The main VOSviewer analytical units, thresholds, and clustering settings are summarized in **Supplementary Table S1**.

### 2.4 Sensitivity analyses

Sensitivity analyses were performed to evaluate the robustness of the principal knowledge-map findings. First, threshold sensitivity analyses were conducted using the main dual-database dataset (**Supplementary Table S2**). For author keyword co-occurrence, the main minimum occurrence threshold was five and sensitivity analyses used thresholds of four and six. For reference co-citation, the main minimum citation threshold was ten and sensitivity analyses used thresholds of eight and twelve. For cited-source co-citation, the main threshold was twenty citations and sensitivity analyses used thresholds of fifteen and twenty-five. In each run, only the threshold of interest was varied while preprocessing, counting method, thesaurus where applicable, and clustering settings were kept unchanged.

A source-sensitivity analysis was conducted using the WoSCC-only dataset (n = 542) to assess whether inclusion of the 12 PubMed-only records and their OpenAlex-linked metadata materially influenced the principal structures. Author keyword co-occurrence, reference co-citation, and cited-source co-citation analyses were repeated using the same preprocessing procedures, thresholds, counting methods, and relevant clustering settings as the primary analyses (**Supplementary Table S3**).

Additional *post hoc* analyses assessed conceptual coverage and potential pathway-specific retrieval dependence without redefining the primary corpus. Titles, abstracts, and author keywords were examined for mechanobiological concepts not explicitly enumerated as individual retrieval terms, including primary cilia/cilium, integrin-related mechanisms, focal adhesion, compression/compressive loading, hydrostatic pressure, joint instability, shear stress/strain, and matrix/substrate stiffness (**Supplementary Table S4**). Records were also classified according to whether their mechanobiology-related retrieval match was supported by broad process-level terms, channel-specific terms (PIEZO1/2 or TRPV4), or both (**Supplementary Table S5**). Twenty-three records matched channel-specific terms without matching the broader process-level descriptors. These records were retained in the primary corpus but excluded from *post hoc* channel-term-independent sensitivity subsets. Author keyword co-occurrence was repeated in the resulting 531-record dual-database subset, and reference co-citation was repeated within the native WoSCC corpus by comparing the 542-record dataset with a 519-record channel-term-independent subset (**Supplementary Table S8**).

Because cluster partitions can depend on VOSviewer parameters, clustering-parameter sensitivity analyses were performed for author keyword co-occurrence and reference co-citation. With item-selection thresholds, preprocessing, thesaurus, and full counting held constant, the clustering resolution was varied from the primary value of 1.00 to 0.75 and 1.25. Small-cluster merging was also disabled at resolution 1.00 while retaining a minimum cluster size of three. Stability was assessed using the number of recovered clusters, item-level correspondence with the primary solution, the adjusted Rand index (ARI), and normalized mutual information (NMI). Because numerical cluster labels are arbitrary across VOSviewer runs, interpretation was based on correspondence of cluster membership rather than cluster numbering (**Supplementary Table S7**).

### 2.5 Keyword–reference correspondence and temporal analyses

For the keyword–reference cluster correspondence analysis, each publication was represented by binary membership indicators for the four author keyword clusters (K1–K4) and four reference co-citation clusters (C1–C4). A publication was assigned to a keyword cluster when it contained at least one author keyword belonging to that cluster and to a reference cluster when its cited-reference list contained at least one reference belonging to that cluster. For each of the 16 K–C combinations, correspondence was quantified as the cosine similarity between the two binary document-incidence vectors.

To distinguish selective correspondence from co-occurrence expected because of cluster prevalence and temporal composition, statistical significance was evaluated using 10,000 year-stratified permutations. Within each publication year, complete reference-cluster profiles were randomly reassigned among publications while keyword-cluster profiles remained fixed. Empirical one-sided P values were calculated from the permutation distributions and adjusted across the 16 comparisons using the Benjamini–Hochberg false discovery rate procedure. Ninety-five percent confidence intervals for observed cosine similarities were estimated using 2,000 year-stratified paired bootstrap resamples. Permutation-adjusted excess correspondence was defined as the observed cosine similarity minus the mean cosine similarity of the corresponding permutation null distribution.

Temporal prominence of leading author keywords was evaluated descriptively at the annual level. For keyword k in year y, annual document share was defined as $100\times n_{k}y/D_{y}$, where $n_{k}y$ was the number of corpus publications in year y containing keyword k and $D_{y}$ was the total number of corpus publications in that year. Bubble size represents annual document frequency and fill represents annual document share.

Temporal changes in the use of the reference-based intellectual structure were evaluated using an annual citation-opportunity-adjusted analysis restricted to the 542 native WoSCC publications while retaining the fixed set of 179 cited references and their original C1–C4 assignments from the primary co-citation network. For each citing year y and cluster c, $D_{y}$ denoted the number of native WoSCC corpus publications in year y, $A_{c}y$ the number of fixed cluster-c references already published and available for citation by year y, and $L_{c}y$ the number of citation links from year-y publications to those available references. Citation opportunity was defined as $D_{y}\times A_{c}y$, and the opportunity-adjusted citation rate was calculated as $R_{c}y=1,000\times L_{c}y/(D_{y}\times A_{c}y)$. An opportunity-adjusted index was then calculated as $OAI_{c}y=R_{c}y/R_{y}$,where $R_{y}$ was the overall annual citation rate across all C1–C4 references available in year y. OAI = 1 therefore represents the annual corpus-wide baseline. The former fixed time-slice analysis and stage-based Shannon and HHI measures were not used for temporal inference. Full annual calculation components are reported in **Supplementary Table S9**; values for 2026 were interpreted cautiously because the publication year was incomplete.

### 2.6 Construction of the literature-informed framework

To synthesize the bibliometric findings, we constructed a literature-informed framework linking mechanical and biomechanical inputs, cellular mechanosensing, inflammatory joint responses, whole-joint remodeling, and future research priorities. The framework was anchored in the principal reference co-citation and author keyword structures, validated keyword–reference correspondences, and opportunity-adjusted temporal patterns. Specific cellular and signaling relationships used to connect these literature-level structures were informed by the cited OA literature rather than inferred as causal relationships from the bibliometric analyses. Translational elements were formulated as hypothesis-generating research priorities requiring prospective experimental and clinical validation.

Mechanical and biomechanical inputs were derived mainly from keyword themes related to mechanical loading, mechanical stress, joint instability, traumatic injury, obesity-related biomechanical burden, and altered matrix stiffness. Cellular mechanosensing and mechanotransduction were derived from clusters and representative terms related to chondrocytes, synoviocytes, osteoblast-lineage cells, Piezo1/Piezo2, TRPV4, integrins, primary cilia, cytoskeletal remodeling, and extracellular matrix-associated signaling. The inflammatory joint microenvironment was summarized from terms and references related to synovitis, monocyte/macrophage activation, cytokines, chemokines, oxidative stress, and inflammasome signaling. Tissue-level OA remodeling was interpreted from themes related to cartilage degeneration, matrix degradation, subchondral bone remodeling, synovial activation, pain-related inflammatory signaling, and whole-joint tissue crosstalk.

On this basis, future research priorities were organized around mechanical-inflammatory phenotyping, integrated imaging and biomarker assessment, patient stratification, mechanically relevant experimental models, modulation of mechanosensitive pathways, and combined mechanical and anti-inflammatory approaches. These elements were included as literature-informed hypotheses for future investigation rather than patient-level findings or validated therapeutic recommendations. **Figure 2** was created using BioGDP to present this synthesis (34). This framework represents a literature-derived interpretation of bibliometric and knowledge-map findings rather than a causal model validated by experimental or clinical data.

**Figure 2:**
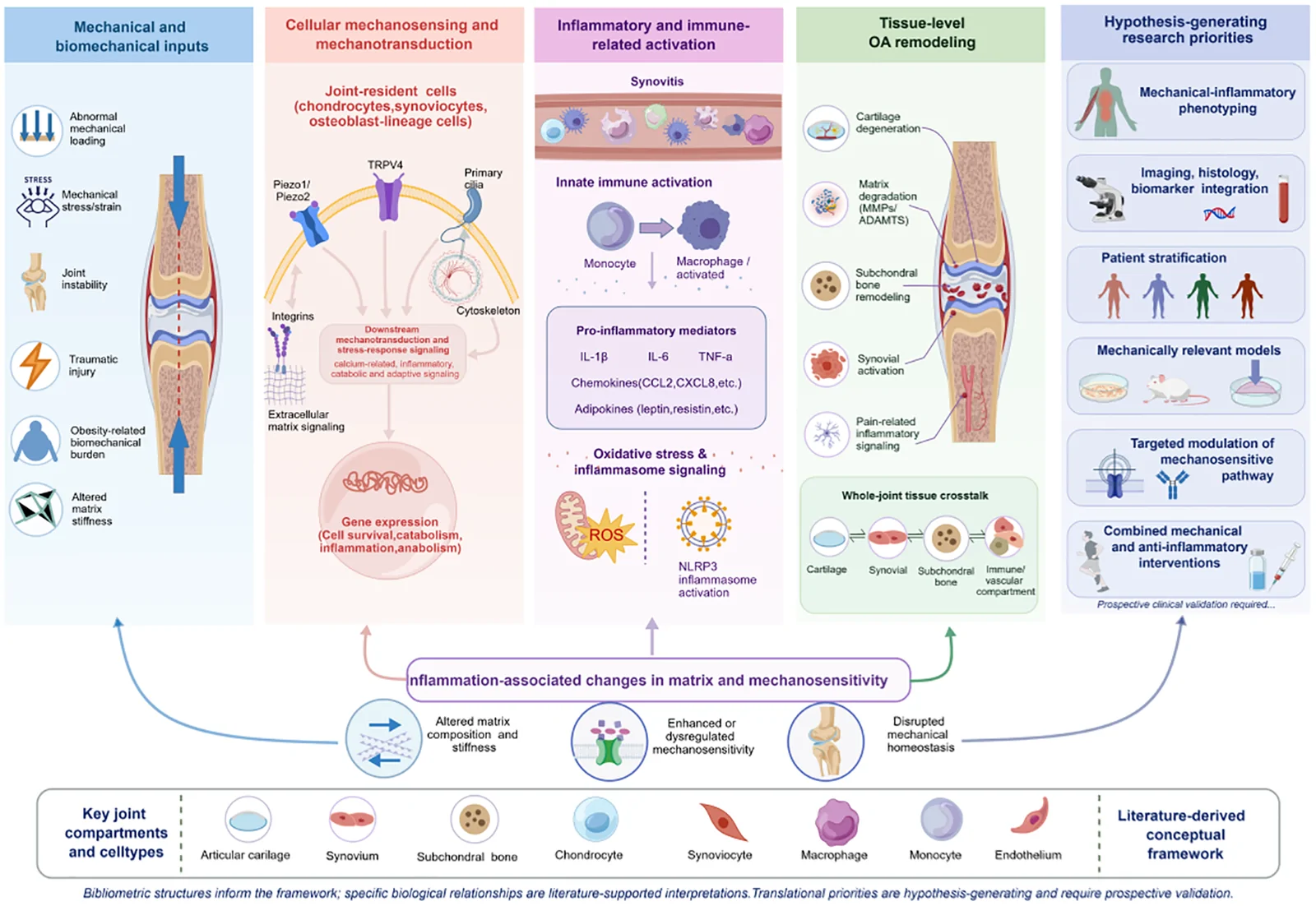
Literature-informed framework linking osteoarthritis mechanoinflammation with whole-joint remodeling and hypothesis-generating research priorities. The framework integrates the principal bibliometric structures identified by reference co-citation, author keyword co-occurrence, validated keyword–reference correspondence, and citation-opportunity-adjusted temporal analysis with biological relationships supported by the cited OA literature. Mechanical and biomechanical inputs are linked conceptually with cellular mechanosensing and mechanotransduction, inflammatory joint responses, and whole-joint tissue remodeling. Specific cellular and signaling relationships shown in the framework represent literature-supported mechanistic interpretation rather than causal relationships established by the bibliometric analyses. The rightmost component summarizes hypothesis-generating research priorities, including mechanical-inflammatory phenotyping, multimodal biomarker assessment, patient stratification, mechanically relevant models, modulation of mechanosensitive pathways, and combined mechanical and anti-inflammatory approaches; these priorities require prospective experimental and clinical validation. Clinical examples involving malalignment, gait, or instability are most directly applicable to knee OA. Created with BioGDP.com (Agreement number: GDP2026CHT24A).

## 3 Results

### 3.1 Publication growth and annual trends

Publication output increased markedly over the study period, with the clearest expansion occurring in the later years of the corpus (**Figure 3**). A total of 554 publications were included. Annual output increased from 2 publications in 2000 to 87 in 2025, followed by 63 publications in 2026. Because the search was conducted on May 23, 2026, the 2026 value represents an incomplete publication year. Before 2013, annual output generally remained below 10 publications per year; from 2014 onward, output increased more steadily, with a pronounced expansion after 2018.

**Figure 3:**
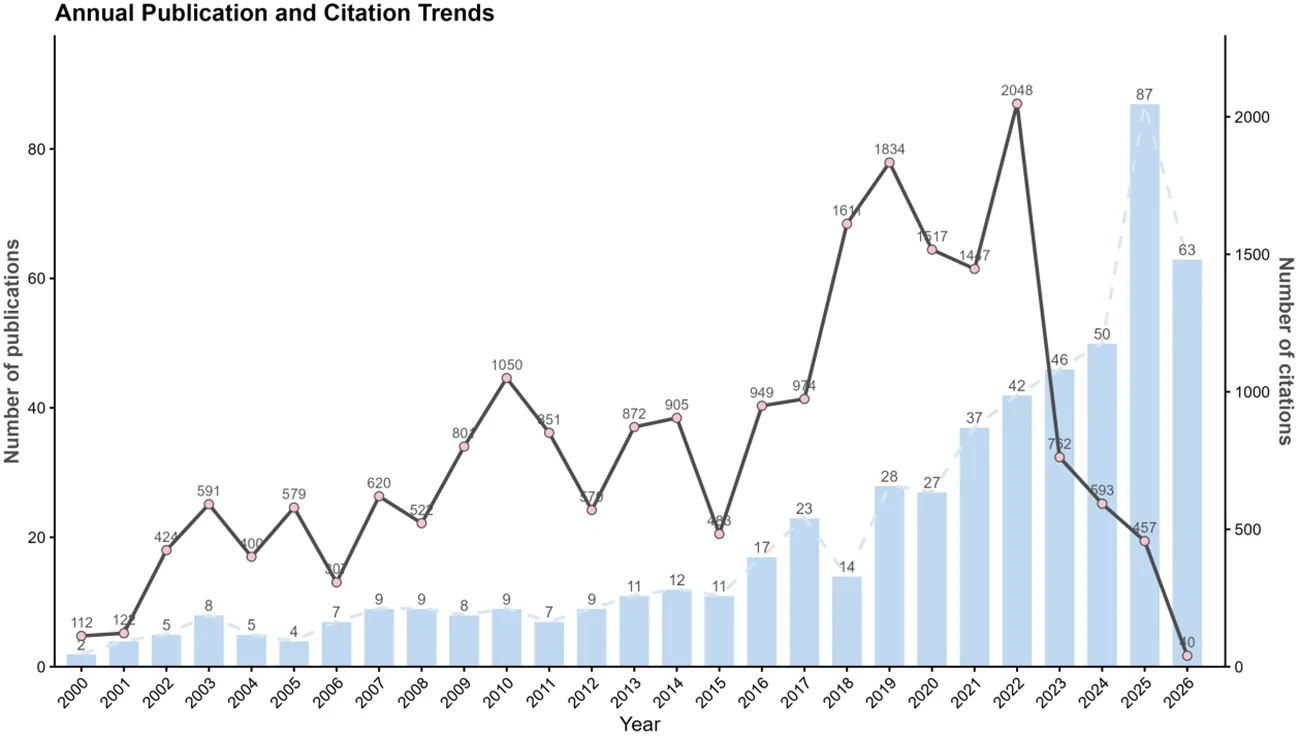
Annual publication output and citation trends from 2000 to 2026. Bars indicate the number of publications per year, the dashed line shows the annual publication trend, and the solid line with circular markers indicates annual citation counts. The left y-axis represents publication output, and the right y-axis represents annual citation counts. Data for 2026 represent an incomplete publication year because the literature search was conducted on May 23, 2026.

Annual citation counts also increased over time but were less linear than publication output. The highest annual citation count was observed in 2022 (2,048 citations), followed by 2019 (1,834) and 2018 (1,611). Citation counts were lower in the most recent years, consistent with shorter citation-accumulation time.

### 3.2 Contributions and collaboration networks

At the country/region level, China contributed the largest number of publications, with 177 records, followed by the United States with 148 records and Japan with 51 records (**Figures 4A, B**; **Table 1**). The United Kingdom ranked fourth with 48 publications. The United States had the highest total citations among the leading countries/regions, with 9,565 citations and a CPP of 64.6, followed by China with 4,261 citations and a CPP of 24.1. France, Spain, South Korea, and Italy also showed relatively high CPP values among the top 15 countries/regions.

**Figure 4:**
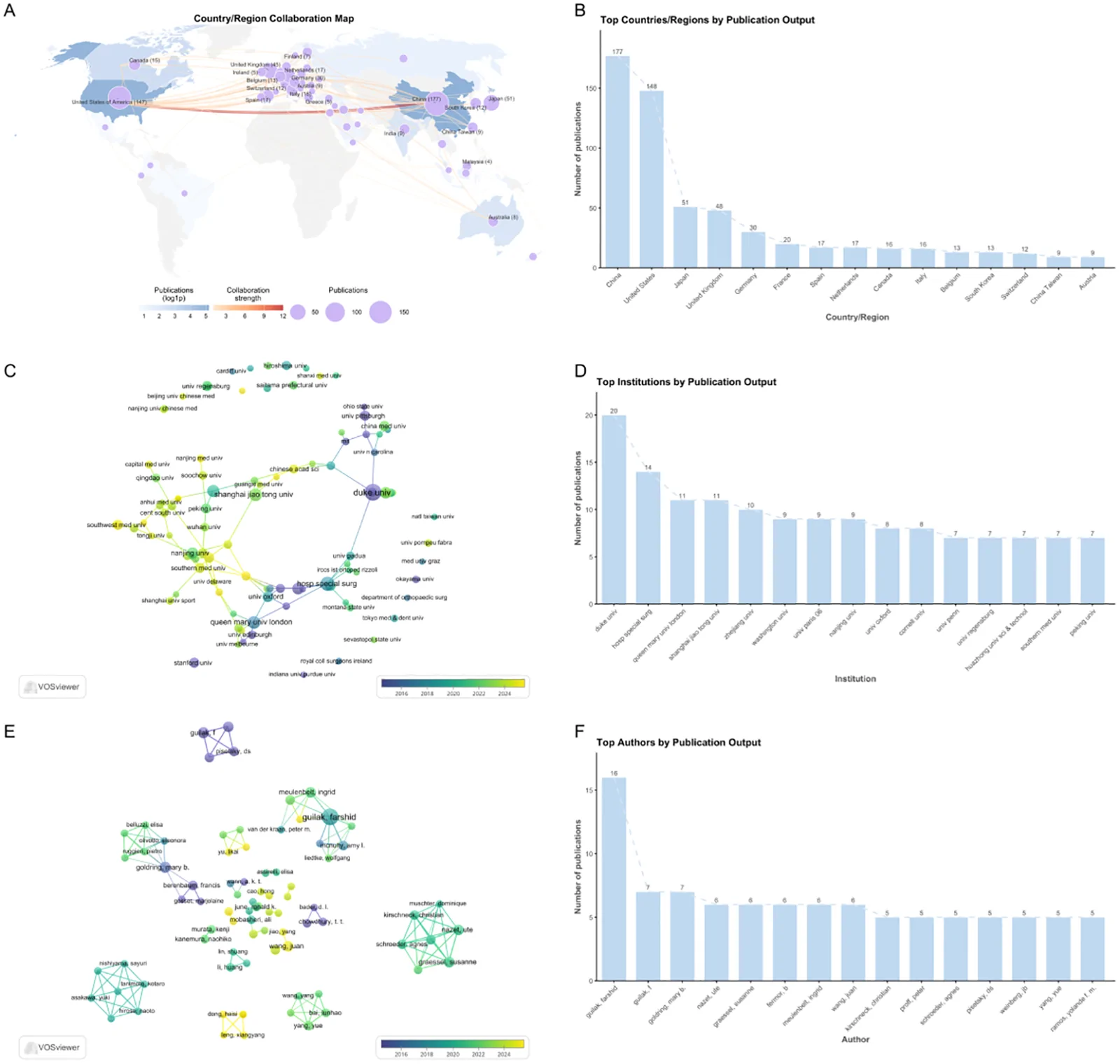
Contributions and collaboration patterns. **(A)** Country/region collaboration map. Circle size represents publication output, color intensity indicates log-transformed publication volume, and connecting lines indicate international collaboration strength. **(B)** Top 15 countries/regions ranked by publication output. **(C)** Institutional collaboration network. Node size represents publication output, links indicate collaboration, and node color represents average publication year. **(D)** Top 15 institutions ranked by publication output. **(E)** Author collaboration network. Node size represents publication output, links indicate co-authorship, and node color represents average publication year. **(F)** Top 15 authors ranked by publication output. The VOSviewer collaboration networks were constructed using full counting with a minimum publication threshold of three. Network-level quantitative indicators are reported in the Results, and country/region rankings are summarized in **Table 1**.

**Table 1:** Leading countries/regions and international collaboration indicators.

Rank	Country/region	Pub., n	Cit., n	CPP	TLS	TLS/Pub.
1	China	177	4,261	24.1	38	0.21
2	United States	148	9,565	64.6	47	0.32
3	Japan	51	1,327	26	2	0.04
4	United Kingdom	48	2,070	43.1	37	0.77
5	Germany	30	1,196	39.9	18	0.6
6	France	20	1,383	69.2	13	0.65
7	Spain	17	1,157	68.1	16	0.94
8	Netherlands	17	937	55.1	12	0.71
9	Canada	16	187	11.7	15	0.94
10	Italy	16	1,014	63.4	7	0.44
11	Belgium	13	714	54.9	21	1.62
12	South Korea	13	863	66.4	9	0.69
13	Switzerland	12	296	24.7	11	0.92
14	China Taiwan	9	104	11.6	5	0.56
15	Austria	9	219	24.3	4	0.44

Pub., publications; Cit., citations; CPP, citations per publication; TLS, total link strength; TLS/Pub., total link strength per publication. Countries/regions were ranked by publication output. TLS was derived from the country/region co-authorship network and indicates the overall strength of international collaboration links. CPP and TLS/Pub. were calculated as normalized indicators of citation impact and collaboration intensity.

The country/region collaboration network included 47 items, 367 links, and a total link strength (TLS) of 997 (**Figure 4A**). The United States showed the highest TLS among the top 15 countries/regions (TLS = 47), followed by China (TLS = 38) and the United Kingdom (TLS = 37). At the institutional level, the collaboration network included 105 items, 131 links, and a TLS of 182 (**Figures 4C, D**). Duke University, Hospital for Special Surgery, and Queen Mary University of London were among the leading institutions by publication output. At the author level, the co-authorship network included 86 items, 130 links, and a TLS of 360 (**Figures 4E, F**). Farshid Guilak was the most productive author in this field. Compared with the country/region network, institutional and author-level networks showed more dispersed collaboration patterns.

### 3.3 Intellectual base, thematic clusters, and emerging keywords

#### 3.3.1 Reference co-citation clusters

Under the primary VOSviewer configuration (resolution = 1.00), reference co-citation analysis resolved 179 cited references into a four-cluster solution comprising 7,122 links and a total link strength of 13,015 (**Figure 5A**; **Table 2**). These clusters were interpreted as four intellectual domains at the primary analytical resolution.

**Figure 5:**
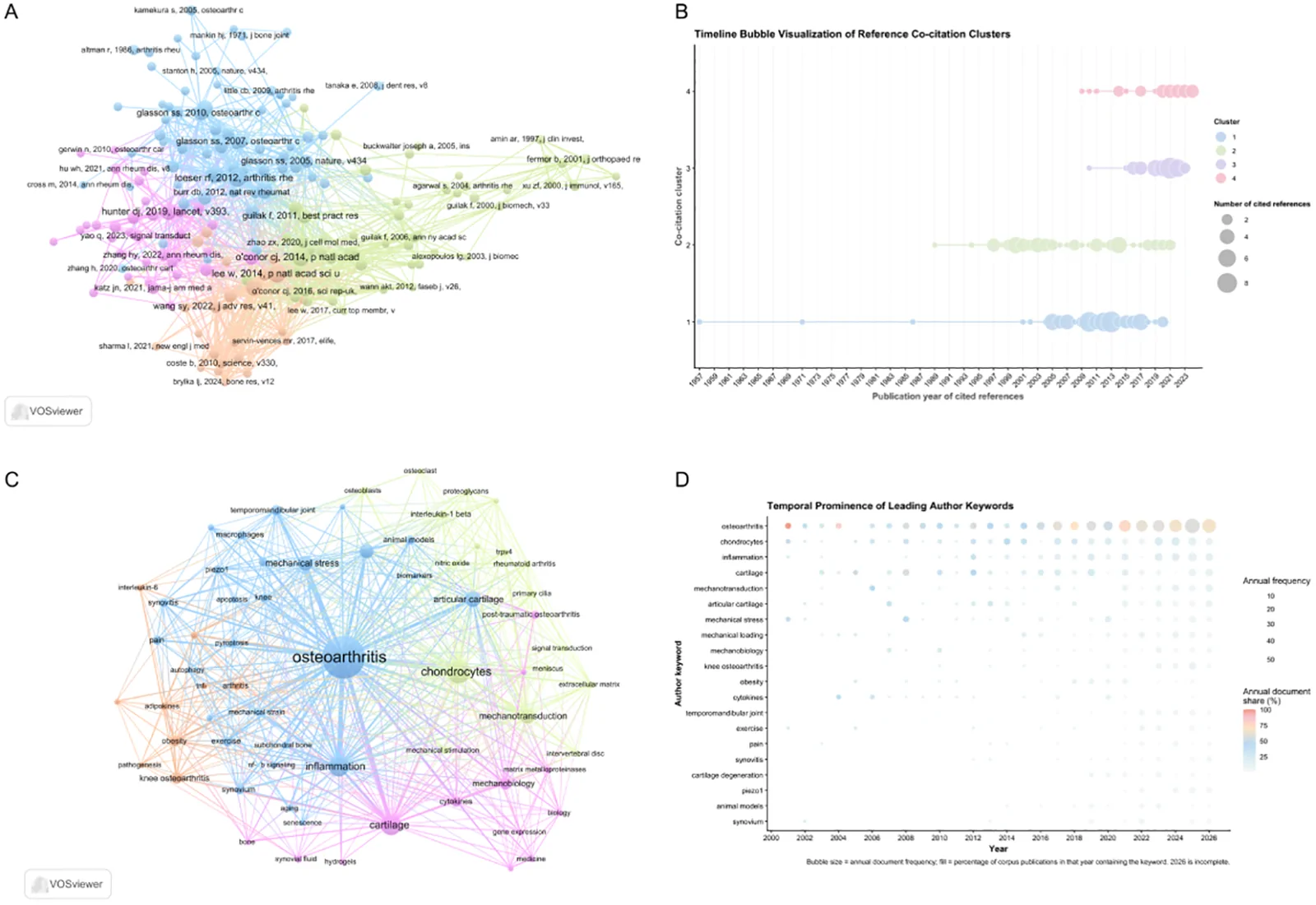
Reference co-citation clusters and author keyword evolution. **(A)** Network visualization of reference co-citation clusters. Each node denotes a cited reference, with larger nodes indicating higher citation frequency. Links represent co-citation relationships, and node colors indicate cluster membership under the primary VOSviewer configuration. **(B)** Timeline bubble plot showing the publication-year distribution of cited references across co-citation clusters. The x-axis shows the publication year of cited references, and bubble size represents the number of cited references in each cluster-year combination. This panel is descriptive and is not used as the citation-opportunity-adjusted temporal analysis. **(C)** Network visualization of author keyword co-occurrence. Each node represents an author keyword, with node size proportional to occurrence frequency. Links indicate keyword co-occurrence, and colors denote thematic clusters under the primary VOSviewer configuration. **(D)** Temporal prominence of the 20 most frequent author keywords. Bubble size represents the annual number of publications containing each keyword, and fill intensity represents the percentage of all corpus publications in the corresponding year containing that keyword. The analysis is descriptive and does not represent formal burst detection. Data for 2026 represent an incomplete publication year. Cluster summaries are provided in **Tables 2**, **3**.

**Table 2:** Main reference co-citation clusters and representative intellectual bases.

Cl.	CRs, n	Cit., n	Rep. refs.	Label	Interpretation
C1	66	1,109	Loeser RF, 2012 (58), Arthritis Rheum; Kapoor M, 2011 (93), Nat Rev Rheumatol; Scanzello CR, 2012 (92), Bone; Glasson SS, 2010 (73), Osteoarthritis Cartilage; Pritzker KPH, 2006 (91), Osteoarthritis Cartilage; Sellam J, 2010 (90), Nat Rev Rheumatol	Classical OA inflammatory pathogenesis and disease models	Classical knowledge base of OA pathogenesis, including cartilage degeneration, synovial inflammation, inflammatory mediators, histological grading, and experimental OA models.
C2	54	785	O’Conor CJ, 2014 (80), PNAS; Guilak F, 2011 (81), Best Pract Res Clin Rheumatol; Phan MN, 2009 (89), Arthritis Rheum; Sanchez-Adams J, 2014 (88), Curr Rheumatol Rep; Zhao ZX, 2020 (63), J Cell Mol Med; Clark AL, 2010 (82), Arthritis Rheum	Mechanical loading and chondrocyte mechanotransduction	Mechanobiological foundation of OA research, focusing on mechanical loading, chondrocyte responses, mechanotransduction, cartilage matrix regulation, and mechanically mediated inflammatory or catabolic responses.
C3	35	526	Hunter DJ, 2019 (87), Lancet; Zhen GH, 2021 (86), Nat Commun; Hodgkinson T, 2022 (85), Nat Rev Rheumatol; Sanchez-Lopez E, 2022 (10), Nat Rev Rheumatol; Zhang HY, 2022 (83), Ann Rheum Dis; Katz JN, 2021 (84), JAMA	Contemporary OA pathogenesis, aging, and tissue crosstalk	Recent conceptual frameworks of OA, emphasizing disease burden, aging-related mechanisms, subchondral bone-cartilage interactions, inflammatory tissue crosstalk, and emerging therapeutic perspectives.
C4	24	400	Lee W, 2014 (59), PNAS; Lee W, 2021 (22), PNAS; Wang SY, 2022 (35), J Adv Res; Gao WN, 2022 (29), Front Cell Dev Biol; Coste B, 2010, Science; Hwang HS, 2015, Int J Mol Sci	Mechanosensitive ion channels and mechanical-injury signaling	Emerging mechanosensitive-channel knowledge base, especially Piezo-related mechanotransduction, mechanically induced calcium signaling, mechanical injury responses, and their links to cartilage degeneration and inflammation.

Cl., cluster; CRs, cited references; Cit., citations; Rep. refs., representative co-cited references; OA, osteoarthritis; PNAS, Proceedings of the National Academy of Sciences of the United States of America. Representative references were selected according to citation frequency and relevance within each reference co-citation cluster.

C1 was the largest cluster, with 66 cited references and 1,109 citations. This cluster was interpreted as classical OA inflammatory pathogenesis and disease models, covering cartilage degeneration, synovial inflammation, inflammatory mediators, histological grading, and experimental OA models. C2 included 54 cited references and 785 citations, and represented mechanical loading and chondrocyte mechanotransduction, with emphasis on mechanical-load responses, cartilage matrix regulation, and mechanically mediated inflammatory or catabolic responses. C3 included 35 cited references and 526 citations, and was interpreted as contemporary OA pathogenesis, aging, and tissue crosstalk. C4 included 24 cited references and 400 citations, and represented mechanosensitive ion channels and mechanical-injury signaling, especially Piezo-related mechanotransduction and mechanically induced calcium signaling.

The timeline bubble plot showed distinct temporal distributions across clusters (**Figure 5B**). Older cited references were mainly concentrated in foundational OA pathogenesis and disease-model research, whereas more recent cited references increasingly involved contemporary OA mechanisms, tissue crosstalk, and mechanosensitive-channel signaling.

#### 3.3.2 Author keyword clusters and temporal keyword patterns

Author keyword co-occurrence analysis identified 66 keywords with at least five occurrences, forming a four-cluster solution under the primary VOSviewer configuration (resolution = 1.00), with 560 links and a total link strength of 1,510 (**Figure 5C**; **Table 3**). For interpretive purposes, these clusters were characterized as four thematic domains within the primary analytical configuration.

**Table 3:** Main author keyword clusters and thematic domains.

Cl.	KWs, n	Occ., n	TLS	Rep. KWs	Label	Interpretation
K1	27	732	1,614	osteoarthritis; inflammation; articular cartilage; mechanical stress; mechanical loading; temporomandibular joint; exercise; cartilage degeneration; pain; synovitis; Piezo1; animal models	Mechanically induced inflammatory degeneration in OA	This cluster represents the broadest thematic domain, linking mechanical stress and loading with OA-related inflammation, cartilage degeneration, synovitis, pain, and emerging mechanosensitive mechanisms such as Piezo1.
K2	17	254	718	chondrocytes; mechanotransduction; interleukin-1 beta; mechanical stimulation; osteoblasts; TRPV4; extracellular matrix; meniscus; tissue engineering; proteoglycans; primary cilia	Chondrocyte mechanotransduction and matrix homeostasis	This cluster focuses on cellular mechanotransduction, especially chondrocyte responses to mechanical stimulation, matrix regulation, TRPV4-related signaling, primary cilia, and extracellular matrix homeostasis.
K3	13	181	476	cartilage; mechanobiology; cytokines; post-traumatic osteoarthritis; synovial fluid; matrix metalloproteinases; metabolism; bone; biology; gene expression; hydrogels	Cartilage mechanobiology and cytokine-mediated matrix remodeling	This cluster emphasizes cartilage-centered mechanobiology, cytokine-related inflammatory responses, matrix metalloproteinase activity, post-traumatic OA, synovial fluid, and tissue remodeling.
K4	9	96	212	knee osteoarthritis; obesity; arthritis; biomechanics; adipokines; interleukin-6; metabolic syndrome; pathogenesis; TNF-α	Metabolic inflammation and biomechanical risk in knee OA	This cluster reflects the intersection of knee OA, obesity, metabolic inflammation, adipokine-related mechanisms, inflammatory cytokines, and biomechanical risk factors.

Cl., cluster; KWs, author keywords; Occ., total occurrences; TLS, total link strength; Rep. KWs, representative author keywords; OA, osteoarthritis; TRPV4, transient receptor potential vanilloid 4; TNF-α, tumor necrosis factor-alpha. Representative keywords were selected mainly according to occurrence frequency within each author keyword cluster. Cluster-level TLS represents the sum of node-level TLS values within each cluster and is not directly comparable with the whole-network total link strength.

K1 was the largest cluster, with 27 keywords and 732 total occurrences. It was interpreted as mechanically induced inflammatory degeneration in OA, linking mechanical stress and loading with inflammation, cartilage degeneration, synovitis, pain, and Piezo1-related mechanisms. K2 contained 17 keywords and 254 occurrences. It represented chondrocyte mechanotransduction and matrix homeostasis, with emphasis on chondrocyte responses, mechanical stimulation, TRPV4, extracellular matrix, proteoglycans, and primary cilia. K3 contained 13 keywords and 181 occurrences. It was interpreted as cartilage mechanobiology and cytokine-mediated matrix remodeling, involving post-traumatic OA, synovial fluid, matrix metalloproteinases, gene expression, and tissue remodeling. K4 contained 9 keywords and 96 occurrences. It represented metabolic inflammation and biomechanical risk in knee OA, linking obesity, biomechanics, adipokines, interleukin-6, metabolic syndrome, and TNF-α.

The keyword timeline showed that broad terms such as osteoarthritis, inflammation, cartilage, and chondrocytes remained recurrent across the corpus, while mechanism-specific terms including mechanotransduction, Piezo1, TRPV4, synovitis, matrix remodeling, and post-traumatic OA were more frequently represented in recent years (**Figure 5D**).

### 3.4 Keyword–reference correspondence and temporal use of intellectual bases

Five K–C pairs showed positive correspondence significantly exceeding the year-stratified null expectation (**Figures 6A, C**; **Table 4**). The largest permutation-adjusted excess was observed for K2–C2 (observed cosine = 0.524, 95% CI 0.465–0.577; null mean = 0.379; excess = 0.145; q < 0.001), linking chondrocyte mechanotransduction and matrix homeostasis with the reference-based knowledge base of mechanical loading and chondrocyte mechanotransduction. K2 also showed significant correspondence with C4 (observed cosine = 0.390, 95% CI 0.322–0.455; null mean = 0.277; excess = 0.114; q < 0.001). K3 showed significant correspondence with C2 (observed cosine = 0.416; excess = 0.076; q = 0.012) and C4 (observed cosine = 0.276; excess = 0.067; q = 0.035), while K1–C3 also remained significant after FDR correction (observed cosine = 0.594; excess = 0.035; q = 0.028). Although K1–C1 had the highest unadjusted cosine similarity (0.704), its value was close to the year-stratified null expectation (approximately 0.701; q approximately 0.892), indicating that the large raw overlap was primarily prevalence-driven rather than selectively enriched. Complete results for all 16 comparisons are provided in **Supplementary Table S10**.

**Figure 6:**
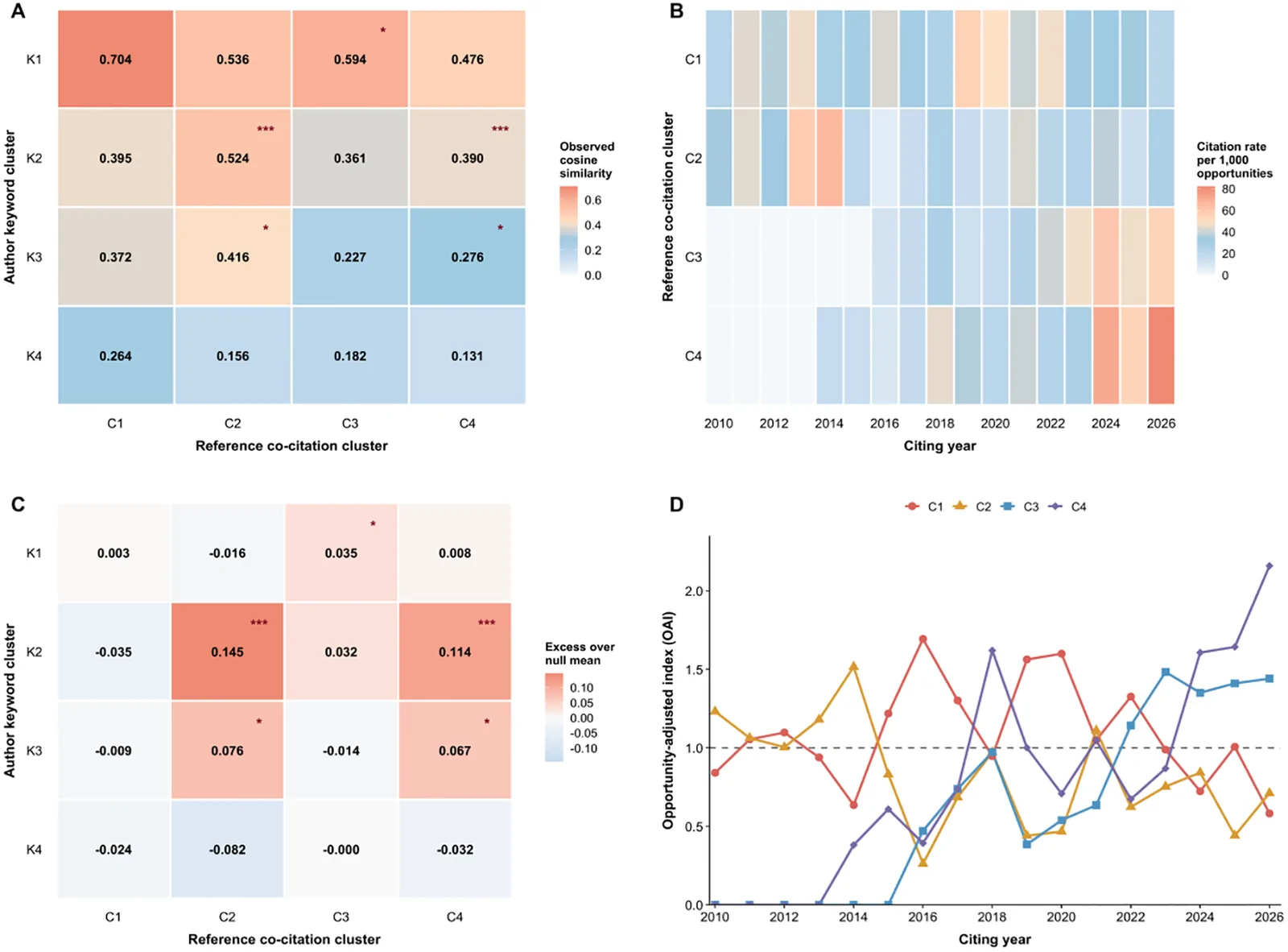
Validated keyword–reference correspondence and citation-opportunity-adjusted temporal dynamics of the reference-based intellectual structure. **(A)** Observed cosine correspondence between author keyword clusters (K1–K4) and reference co-citation clusters (C1–C4), calculated from binary document-incidence profiles. Asterisks indicate statistical significance based on 10,000 year-stratified permutations with Benjamini–Hochberg false discovery rate correction across all 16 K–C comparisons (*q < 0.05, **q < 0.01, ***q < 0.001). **(B)** Annual opportunity-adjusted citation rate of the four reference co-citation clusters, expressed as citation links per 1,000 citation opportunities. The analysis used the 542 native WoSCC citing publications while retaining the fixed primary set of 179 cited references and their original C1–C4 assignments. **(C)** Permutation-adjusted excess correspondence for all K–C combinations, defined as the observed cosine similarity minus the mean cosine similarity from the corresponding year-stratified permutation null distribution. Asterisks indicate FDR-significant positive correspondence. **(D)** Annual opportunity-adjusted index (OAI) trajectories for reference co-citation clusters C1–C4. OAI = 1 represents the overall annual citation-rate baseline across all C1–C4 references available for citation in that year; values above 1 indicate above-baseline use after adjustment for citation opportunity. The 2026 estimates are shown for completeness but should be interpreted cautiously because the publication year was incomplete at the retrieval date.

**Table 4:** Validated correspondence between author keyword clusters and reference co-citation clusters.

Pair	Observed cosine	95% bootstrap CI	Null mean	Excess	Docs, n	FDR q
K2–C2	0.524	0.465–0.577	0.379	0.145	105	<0.001
K2–C4	0.390	0.322–0.455	0.277	0.114	62	<0.001
K3–C2	0.416	0.353–0.472	0.340	0.076	74	0.012
K3–C4	0.276	0.211–0.344	0.210	0.067	39	0.035
K1–C3	0.594	0.551–0.635	0.558	0.035	173	0.028

Only positive K–C pairs significant after Benjamini–Hochberg FDR correction across all 16 comparisons are shown. Observed cosine was calculated from binary document-incidence vectors. Null mean is the mean cosine from 10,000 year-stratified permutations. Excess is observed cosine minus the permutation null mean. The 95% CI was estimated using 2,000 year-stratified paired bootstrap resamples. Docs, n is the number of publications assigned to both clusters under the validated document-level definition. All 16 pairwise results are provided in **Supplementary Table S10**.

The opportunity-adjusted analysis showed substantial year-to-year variation in the reference-based intellectual structure (**Figures 6B, D**; **Table 5**; **Supplementary Table S9**). C1 and C2 contributed strongly to earlier citation activity, but their relative use varied across years. In recent years, C3 remained above the annual baseline from 2022 through 2025 (OAI = 1.142, 1.483, 1.351, and 1.410, respectively), whereas C4 showed marked above-baseline use in 2024 and 2025 (OAI = 1.606 and 1.643). C1 was near the annual baseline in 2023 and 2025 but below it in 2024, while C2 remained below baseline during 2022–2025. The 2026 estimates (C3 = 1.440; C4 = 2.158) are reported for completeness but represent an incomplete publication year.

**Table 5:** Annual opportunity-adjusted citation index of reference co-citation clusters.

Citing year	C1	C2	C3	C4
2010	0.841	1.231	0	0
2011	1.053	1.062	0	0
2012	1.097	1.003	0	0
2013	0.939	1.179	0	0
2014	0.635	1.516	0	0.381
2015	1.218	0.831	0	0.609
2016	1.693	0.262	0.471	0.392
2017	1.302	0.685	0.738	0.738
2018	0.946	0.972	0.972	1.62
2019	1.562	0.44	0.385	1
2020	1.599	0.467	0.539	0.708
2021	1.049	1.11	0.634	1.047
2022	1.326	0.623	1.142	0.673
2023	0.988	0.753	1.483	0.868
2024	0.723	0.841	1.351	1.606
2025	1.006	0.441	1.41	1.643
2026*	0.582	0.711	1.44	2.158

Values are opportunity-adjusted citation indices (OAI). For each citing year, OAI = 1 represents the overall annual citation rate across all C1–C4 references available for citation in that year. Values >1 indicate above-baseline use after accounting for the number of citing documents and available cluster references. Full annual calculation components are provided in **Supplementary Table S9**. *2026 was incomplete at the retrieval date.

### 3.5 Journal landscape and cross-disciplinary knowledge flows

Journal-level analyses were performed to characterize publication outlets, cited-source structure, and cross-disciplinary citation flows (**Figure 7**; **Table 6**). In the bibliographic coupling network of source journals, journals with at least three documents were included. The network contained 47 journals, 3 clusters, 958 links, and a total link strength of 13,017 (**Figure 7A**).

**Figure 7:**
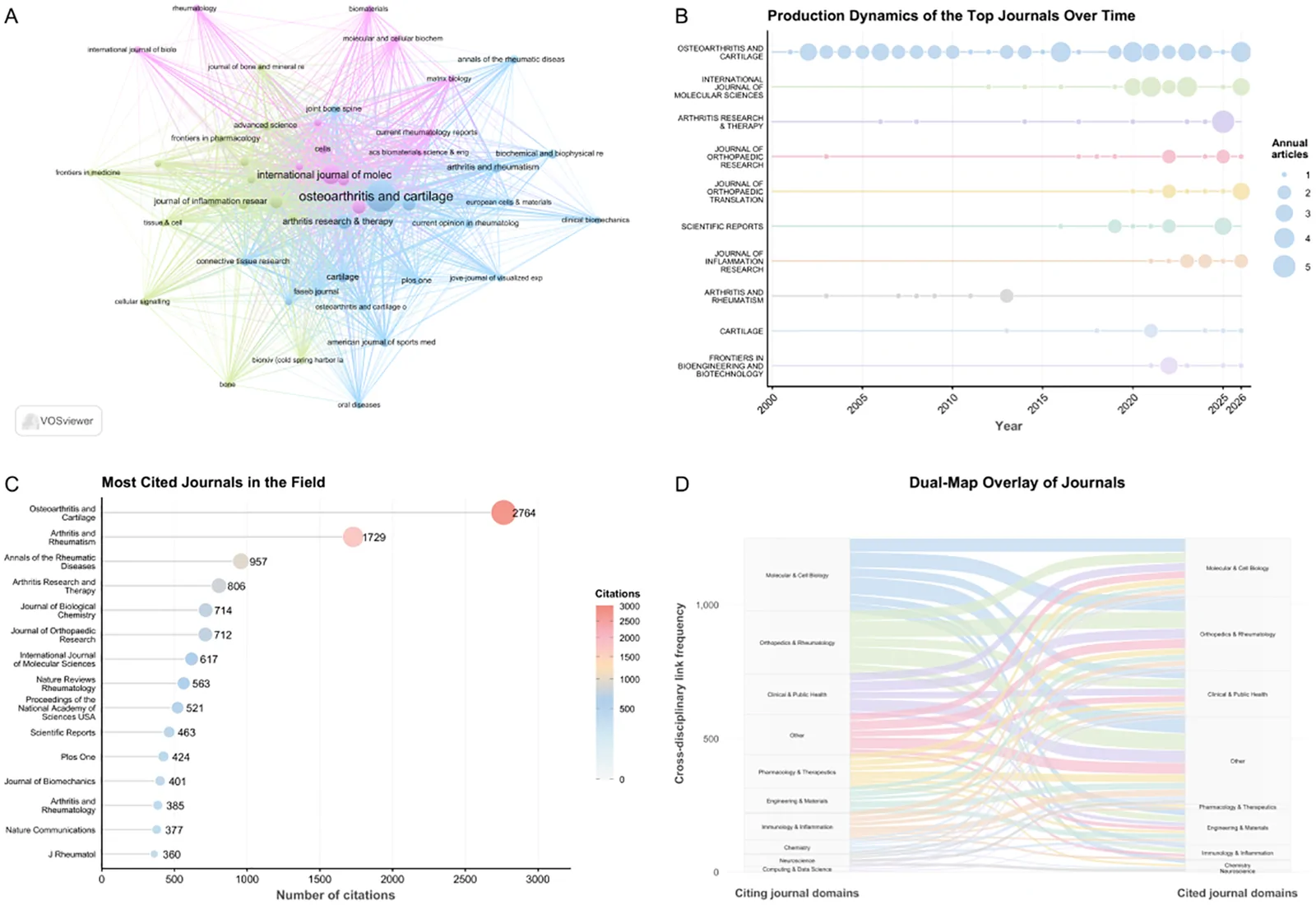
Journal landscape and cross-disciplinary knowledge flows. **(A)** Bibliographic coupling network of source journals. Nodes represent journals, node size indicates publication output, links indicate bibliographic coupling relationships, and colors represent journal clusters. The network was generated using full counting with a minimum threshold of three documents per source and a minimum cluster size of 3. A total of 47 journals were included, forming 3 clusters, 958 links, and a total link strength of 13,017. **(B)** Production dynamics of the top journals over time. Bubble size represents the number of articles published by each journal in a given year. **(C)** Most cited journals in the field based on cited-source analysis. Bubble size and color intensity represent the number of citations. **(D)** Dual-map overlay of journals showing cross-disciplinary knowledge flows between citing and cited journal domains. The left side represents citing journal domains, and the right side represents cited journal domains. Flow width represents cross-disciplinary link frequency. The image summarizes the publication outlets, cited-source structure, and cross-disciplinary journal-domain relationships in mechanobiology-related inflammatory research in osteoarthritis.

**Table 6:** Leading source journals, cited journals, and journal-domain citation paths.

Panel A Top active source journals			
Rank	Source journal	Pub., n	Active yrs
1	*Osteoarthritis and Cartilage*	54	2001–2026
2	*International Journal of Molecular Sciences*	21	2012–2026
3	*Arthritis Research & Therapy*	12	2006–2025
4	*Journal of Orthopaedic Translation*	10	2020–2026
5	*Journal of Orthopaedic Research*	10	2003–2026
6	*Scientific Reports*	10	2016–2025
7	*Journal of Inflammation Research*	9	2021–2026
8	*Frontiers in Bioengineering and Biotechnology*	7	2021–2026
9	*Cartilage*	7	2013–2026
10	*Arthritis and Rheumatism*	7	2003–2013

Panel B. Top cited journals			
Rank	Cited journal	Cit., n	
1	*Osteoarthritis and Cartilage*	2,764	
2	*Arthritis and Rheumatism*	1,729	
3	*Annals of the Rheumatic Diseases*	957	
4	*Arthritis Research & Therapy*	806	
5	*Journal of Biological Chemistry*	714	
6	*Journal of Orthopaedic Research*	712	
7	*International Journal of Molecular Sciences*	617	
8	*Nature Reviews Rheumatology*	563	
9	*Proceedings of the National Academy of Sciences USA*	521	
10	*Scientific Reports*	463	

Panel C. Main journal-domain citation paths			
Rank	Main citation path	Weight	Share
1	Orthopedics & Rheumatology to Orthopedics & Rheumatology	3,802	9.40%
2	Molecular & Cell Biology to Other	3,792	9.40%
3	Orthopedics & Rheumatology to Other	3,306	8.20%
4	Molecular & Cell Biology to Orthopedics & Rheumatology	3,120	7.70%
5	Molecular & Cell Biology to Molecular & Cell Biology	2,554	6.30%
6	Clinical & Public Health to Other	1,628	4.00%
7	Other to Other	1,479	3.70%
8	Clinical & Public Health to Orthopedics & Rheumatology	1,401	3.50%
9	Orthopedics & Rheumatology to Molecular & Cell Biology	1,395	3.50%
10	Other to Orthopedics & Rheumatology	1,205	3.00%

Pub., publications; Active yrs, active publication years; Cit., citations; Weight, citation-path weight; Share, percentage of the corresponding citation path. Panel A ranks source journals by publication output, Panel B ranks cited journals by cited-source frequency, and Panel C ranks journal-domain citation paths by citation-path weight.

Osteoarthritis and Cartilage was the most active source journal, with 54 publications from 2001 to 2026 (**Table 6**). It was followed by International Journal of Molecular Sciences with 21 publications, Arthritis Research & Therapy with 12 publications, and Journal of Orthopaedic Translation, Journal of Orthopaedic Research, and Scientific Reports, each with 10 publications. In the cited-source analysis, Osteoarthritis and Cartilage also ranked first, with 2,764 cited-source counts (**Figure 7C**; **Table 6**). Other leading cited journals included Arthritis and Rheumatism (1,729), Annals of the Rheumatic Diseases (957), Arthritis Research & Therapy (806), Journal of Biological Chemistry (714), and Journal of Orthopaedic Research (712).

The dual-map overlay showed cross-disciplinary citation flows among orthopedics, rheumatology, molecular and cellular biology, clinical and public health, and other journal domains (**Figure 7D**; **Table 6**). The strongest path was from Orthopedics & Rheumatology to Orthopedics & Rheumatology, with a weight of 3,802 and a share of 9.40%. Other major paths included Molecular & Cell Biology to Other (weight = 3,792, 9.40%), Orthopedics & Rheumatology to Other (weight = 3,306, 8.20%), and Molecular & Cell Biology to Orthopedics & Rheumatology (weight = 3,120, 7.70%).

### 3.6 Sensitivity analyses of threshold selection and database source

*Post hoc* corpus and retrieval-dependency analyses provided additional checks on the scope of the original search strategy. A corpus-coverage audit showed that mechanobiological concepts not explicitly enumerated as individual retrieval terms were nevertheless represented within the final corpus, including compression/compressive loading (n = 65), shear stress/strain (n = 22), integrin-related mechanisms (n = 18), primary cilia/cilium (n = 14), joint instability (n = 9), hydrostatic pressure (n = 8), matrix/substrate stiffness (n = 6), and focal adhesion (n = 3). Overall, 127 of the 554 publications (22.9%) contained at least one of these concepts in their titles, abstracts, or author keywords (**Supplementary Table S4**).

Twenty-three publications (4.15% of the primary corpus) matched channel-specific PIEZO1/2- or TRPV4-related retrieval terms without matching the broader process-level descriptors. Excluding these records yielded a 531-publication subset for author keyword analysis. The network retained four clusters, with 64 of the original 66 keyword nodes remaining above the occurrence threshold; 62 of the 64 shared keywords (96.9%) retained their cluster assignment (ARI = 0.930; NMI = 0.914). TRPV4 and osteoclast fell below the threshold, while only hyaluronic acid and synovial fluid changed cluster membership (**Supplementary Table S8**).

Within the native WoSCC corpus, exclusion of the same 23 records yielded a 519-publication subset for reference co-citation analysis. The four-cluster solution was retained, with 167 references remaining above the ≥10-citation threshold compared with 177 in the full WoSCC dataset. Among the 167 shared references, 164 (98.2%) retained their cluster assignment (ARI = 0.952; NMI = 0.935). Twenty-three of the 24 original C4 references remained above threshold and all 23 retained C4 membership (**Supplementary Table S8**).

The source-sensitivity analysis showed that 542 of the 554 publications were represented by native WoSCC records and 12 were unique to PubMed. All 12 PubMed-only records had unique exact-DOI OpenAlex matches with concordant PMID, normalized title, and publication year (**Supplementary Table S6**). In the WoSCC-only reference co-citation analysis, 177 of the 179 primary references remained above threshold, the four-cluster solution was retained, and 174 of 177 shared references (98.3%) retained their cluster assignment (ARI = 0.945; NMI = 0.941). The cited-source structure was also highly stable, whereas smaller author-keyword domains showed greater sensitivity to database composition (**Supplementary Table S3**).

Clustering-parameter sensitivity analyses showed that the exact number and boundaries of clusters depended on the VOSviewer resolution parameter, whereas disabling small-cluster merging had no effect on either principal network (**Supplementary Table S7**). For author keywords, resolution 0.75 produced two broader clusters and resolution 1.25 produced six clusters. For reference co-citation, the corresponding settings produced two and six clusters, respectively. At higher resolution, C3 remained almost entirely intact and C4 was retained in its entirety. The four-cluster solutions therefore provide interpretable intermediate-resolution representations of the networks rather than parameter-invariant partitions.

### 3.7 Literature-informed framework

Based on the reference co-citation and author keyword co-occurrence structures, the validated keyword–reference correspondence analysis, and the citation-opportunity-adjusted temporal analysis, we constructed a literature-informed framework to summarize the principal knowledge structure of mechanobiology-related inflammation in OA (**Figure 2**). The framework organizes these literature-level findings across mechanical and biomechanical inputs, cellular mechanosensing, inflammatory joint responses, whole-joint tissue remodeling, and hypothesis-generating research priorities.

The framework summarizes the principal literature-level relationships identified across the analyses (**Figure 2**). Mechanical and biomechanical inputs and cellular mechanosensing were represented by keyword and co-citation structures involving loading, stress, chondrocytes, Piezo1/Piezo2, TRPV4, integrins, primary cilia, cytoskeletal remodeling, and extracellular matrix-associated signaling. The inflammatory joint microenvironment was represented by themes involving inflammation, synovitis, cytokine signaling, pain, and mechanically induced tissue degeneration. Validated keyword–reference correspondence further linked the chondrocyte mechanotransduction and cartilage mechanobiology themes to the mechanical-loading and mechanosensitive-channel knowledge bases, while the broader mechanically induced inflammatory-degeneration theme showed significant excess correspondence with the contemporary OA pathogenesis, aging, and tissue-crosstalk knowledge base. Tissue-level remodeling was represented by cartilage degeneration, subchondral bone and synovial responses, pain-related signaling, and whole-joint tissue crosstalk. The final component of the framework summarizes hypothesis-generating research priorities rather than validated patient strata or treatment effects.

## 4 Discussion

### 4.1 Principal findings and mechanoinflammatory context

This bibliometric and knowledge-map analysis shows that mechanobiology-related inflammatory research in OA has expanded substantially and now spans several interconnected literatures involving mechanotransduction, mechanosensitive channels, synovial inflammation, tissue crosstalk, and translational research (10, 15, 27, 29). At the same time, the contribution and collaboration analyses showed that the field is supported by multiple productive countries, institutions, authors, and journals, although institutional and author-level networks remain relatively dispersed. This fragmentation may hinder the integration of biomechanics, innate inflammation, stromal-cell biology, imaging, biomarker research, experimental modeling, and clinical translation (35–37).

The validated correspondence analysis further refined the relationship between active keyword themes and reference-based intellectual structures. The strongest selective relationships were concentrated in mechanotransduction-related domains: K2 and K3 showed above-null correspondence with the mechanical-loading/chondrocyte-mechanotransduction knowledge base and the mechanosensitive-channel/mechanical-injury knowledge base, whereas K1 showed a smaller but significant excess correspondence with contemporary OA pathogenesis, aging, and tissue crosstalk. In contrast, the high raw K1–C1 cosine did not exceed its year-stratified null expectation, indicating that broad prevalence can generate substantial descriptive overlap without selective document-level enrichment.

Across co-citation structures, keyword themes, and journal-level knowledge flows, the field is increasingly organized around a shared question: how mechanical conditions in the joint are sensed by resident cells, translated into innate inflammatory and catabolic responses, and further connected to immune–stromal communication, tissue remodeling, and clinical heterogeneity (33, 38, 39). The reference co-citation clusters identified complementary intellectual bases encompassing classical OA inflammatory pathogenesis, mechanical loading and chondrocyte mechanotransduction, contemporary whole-joint pathogenesis, and mechanosensitive ion-channel signaling (8, 19, 40, 41). The keyword clusters further showed that active research is concentrated around mechanically induced inflammatory degeneration, chondrocyte mechanotransduction, cytokine-mediated matrix remodeling, and metabolic-inflammatory risk (9, 42, 43).

The concentration of co-citation around several publications also reflects differences in their roles within the field. Some highly co-cited papers provided widely reused methodological standards or disease-model frameworks, whereas others offered broad conceptual syntheses or experimentally tractable mechanotransduction mechanisms that could be cited across multiple OA subfields (**Table 2**). Their prominence is also influenced by publication age and cross-disciplinary applicability. Accordingly, citation and co-citation prominence in this study are interpreted as indicators of intellectual influence within the literature rather than direct measures of biological importance or study quality.

The opportunity-adjusted temporal analysis indicates a gradual and heterogeneous redistribution of citation attention rather than a discrete stage transition. Earlier citation activity drew heavily on C1 and C2, whereas recent complete years showed greater relative use of C3 and C4 after accounting for both the number of citing publications and the references available for citation. C3 remained above the annual baseline from 2022 to 2025, and C4 showed above-baseline use in 2024 and 2025. These patterns indicate a recent shift in relative citation use toward contemporary OA/tissue-crosstalk and mechanosensitive-channel references, while retaining substantial year-to-year variation.

The knowledge structure identified in this study suggests that OA mechanoinflammation is a context-dependent process shaped by the interaction among mechanical exposure, tissue state, stromal-cell responses, and innate inflammatory activity. Mechanical loading can support joint homeostasis under physiological conditions, whereas abnormal loading, matrix damage, aging-related tissue vulnerability, metabolic stress, and inflammatory priming may change how joint-resident cells and immune cells interpret or respond to mechanical cues (44, 45). In this setting, mechanosensitive channels, extracellular matrix-associated signaling, cytoskeletal remodeling, synovial fibroblast activation, macrophage-related responses, oxidative stress, inflammasome signaling, and matrix-degrading enzymes become components of a connected mechanical-inflammatory process (1, 15, 17, 46). This interpretation helps explain why recent studies increasingly link mechanotransduction with synovitis, subchondral bone remodeling, pain-related inflammatory signaling, and whole-joint tissue crosstalk. An important implication is that the relationship may be bidirectional. Mechanical stress can induce inflammatory and catabolic responses, while inflammatory signaling may alter matrix composition, tissue stiffness, and cellular mechanosensitivity. Such changes may increase subsequent responses to mechanical stimulation and sustain a feed-forward process of inflammation and tissue damage.

These literature-level relationships also provide a basis for considering how mechanoinflammatory processes may contribute to OA heterogeneity. Existing studies indicate that patients with similar radiographic severity may differ in mechanical loading, synovial inflammation, metabolic background, tissue vulnerability, pain mechanisms, and treatment response (47–49). The present analysis does not define patient-level subgroups, but it highlights mechanistic domains that can be examined jointly in future phenotyping and intervention studies. The following sections consider these implications in relation to whole-joint remodeling, mechanosensing, phenotyping, and methodological priorities.

### 4.2 Mechanical-inflammatory responses in whole-joint OA

A central implication of this study is that OA mechanoinflammation can be understood as a multi-level process linking mechanical exposure, cellular mechanosensing, innate inflammatory responses, and tissue remodeling. Abnormal loading, joint instability, traumatic injury, obesity-related biomechanical burden, and altered matrix stiffness may act as upstream pressures on the joint (10, 50). These signals are sensed by joint-resident cells through mechanosensitive ion channels, integrins, primary cilia, cytoskeletal structures, and extracellular matrix-associated pathways, with downstream effects on inflammatory signaling, oxidative stress, inflammasome activation, matrix degradation, synovial responses, and subchondral bone remodeling (51, 52).

**Figure 2** summarizes this mechanical-inflammatory axis within a whole-joint context. The biological effects of mechanical stress depend on the state of the joint microenvironment. Physiological loading can support cartilage homeostasis and matrix turnover, whereas similar mechanical inputs may become pathological in joints characterized by matrix damage, inflammatory priming, cellular senescence, metabolic stress, or altered tissue stiffness (51). Mechanical signals are therefore interpreted through the existing inflammatory and tissue context of the joint (53).

This interpretation is consistent with the whole-joint view of OA. Although cartilage degeneration remains central, OA progression also involves synovium, subchondral bone, meniscus, infrapatellar fat pad, immune cells, and periarticular tissues (1). Mechanical perturbation in one compartment may influence other compartments through inflammatory mediators, extracellular matrix fragments, vascular responses, subchondral bone-cartilage interaction, and pain-related signaling (35). The recent opportunity-adjusted increase in the relative use of the C3 knowledge base is consistent with this broader view.

The shift from a predominantly cartilage-centered view to a whole-joint framework has also broadened how inflammation is understood in OA. The synovium provides a cellular environment in which macrophages, synovial fibroblasts, cytokines, and chemokines can amplify local inflammatory responses; subchondral bone participates through osteochondral remodeling and soluble-factor exchange; and meniscal, adipose, vascular, and other periarticular tissues can contribute additional mechanical and inflammatory signals (1, 10, 35, 54). This broader tissue context shifts attention from isolated immune-cell activity toward reciprocal interactions between immune populations and joint-resident stromal cells across anatomical compartments. The increasing representation of tissue-crosstalk themes in the reference structure is consistent with this whole-joint perspective, while the specific cellular routes connecting these compartments are established by the underlying biological literature rather than by the bibliometric map itself.

The same framework also helps explain the connection between metabolic and biomechanical factors. Obesity-related OA may involve increased joint loading, adipokine signaling, low-grade inflammation, altered metabolism, and tissue vulnerability (55, 56). Post-traumatic OA may involve acute mechanical disruption, matrix damage, inflammatory activation, and later changes in cellular mechanosensitivity (30, 57). Aging-related OA may involve matrix stiffening, cellular senescence, oxidative stress, and reduced adaptive capacity to mechanical load (41, 58). These disease contexts suggest that OA translation requires models that connect mechanical conditions with inflammatory and tissue-level states.

Together, these findings indicate that mechanical exposure, innate inflammatory activity, and tissue state should be evaluated within the same whole-joint framework rather than as separate components of OA pathogenesis.

### 4.3 Mechanosensing pathways at the interface of mechanical stress and inflammation

Within the operationally defined corpus, mechanosensitive ion channels and mechanical-injury signaling formed a distinct reference-based domain, with greater opportunity-adjusted use in the most recent complete years. Piezo1, Piezo2, TRPV4, integrins, primary cilia, and cytoskeletal components provide biologically plausible interfaces through which physical forces are converted into cellular responses (33, 59, 60).

This trend has shifted attention toward more specific mechanotransduction mechanisms with potential translational relevance. Earlier studies often examined how compression, shear stress, loading, or injury affected cartilage matrix degradation and chondrocyte catabolism (61, 62). Recent work has increasingly focused on how physical stimuli are detected, how calcium influx and downstream signaling are activated, how inflammatory mediators are induced, and how matrix-degrading enzymes, oxidative stress, inflammasome signaling, and stress-related cell responses contribute to OA progression (51, 63). These mechanisms are particularly relevant to translation because they may provide measurable links between mechanical exposure, inflammatory activity, and patient phenotype.

Mechanosensitive pathways also connect joint-resident cell responses with innate inflammatory regulation. Although OA is not classically considered an autoimmune disease, inflammatory responses remain central to its progression and symptoms (64). The relevance of OA mechanobiology extends beyond immune-cell infiltration. It also concerns how chondrocytes, synoviocytes, osteoblast-lineage cells, macrophages, endothelial cells, and other joint-associated cells develop inflammatory or catabolic phenotypes under mechanical challenge (54). Mechanical stimulation may influence cytokine production, chemokine release, macrophage-related signaling, synovial activation, matrix metalloproteinase activity, and pain-related mediators. Inflammatory priming may further alter cellular mechanosensitivity and matrix-cell interactions (22, 65). This bidirectional interaction places mechanosensing pathways at the interface between stromal-cell dysfunction and innate inflammatory amplification.

These pathways therefore provide candidate interfaces for future biomarker and intervention studies. Potential directions include mechanically responsive biomarkers, tissue- and context-specific modulation of abnormal mechanosensing, rehabilitation strategies that restore physiological loading, and combined mechanical and anti-inflammatory approaches. Their effects are likely to vary by cell type, tissue compartment, loading condition, and disease stage, and require direct experimental and clinical evaluation.

### 4.4 Hypothesis-generating translational priorities

OA is clinically and biologically heterogeneous, and phenotype- and endotype-based approaches have been proposed to improve disease characterization and therapeutic development (48, 66, 67). The mechanical-inflammatory phenotyping framework considered here has not been validated at the patient level and is therefore best viewed as a hypothesis for prospective investigation rather than an established clinical classification.

One immediate direction is mechanical-inflammatory phenotyping (66, 67). Clinical studies could integrate malalignment, instability, gait, prior injury, muscle weakness, and obesity-related mechanical burden with synovitis, cytokine and chemokine profiles, adipokines, matrix-degradation markers, and pain-related inflammatory mediators. Imaging features, including cartilage loss, bone marrow lesions, effusion-synovitis, and subchondral bone changes, could then be combined with biomarkers and symptoms to test whether reproducible subgroups with different dominant disease processes can be identified (68). If prospectively validated, such profiles could help distinguish patients with predominantly mechanical disease from those with stronger inflammatory or metabolic-inflammatory components. Many candidate mechanical measures in this framework, including malalignment, gait abnormalities, and instability, are most directly applicable to knee OA. Their relevance to hip, hand, or other OA contexts will require joint-specific adaptation and validation rather than direct extrapolation.

A second direction concerns biomarker development. Many OA biomarkers have been studied as general indicators of cartilage degradation, synovial inflammation, or bone remodeling (69, 70). Recent clinical and omics biomarker studies further suggest that biomarker development should move toward integrated diagnostic, prognostic, and treatment-stratification frameworks (36, 68). The present knowledge structure suggests evaluating whether biomarker panels can distinguish mechanically induced inflammatory activity, altered tissue mechanosensitivity, and inflammation-driven remodeling. If prospectively validated, such markers could support disease monitoring, patient selection, and assessment of whether mechanical or anti-inflammatory treatments modify the intended biological process (71).

Experimental models represent another important translational step (21, 72). Mechanical and inflammatory stimuli are often studied separately, although clinical OA develops within a joint environment shaped by inflammatory priming, matrix alteration, aging, and metabolic stress. Future models could combine controlled loading with inflammatory readouts such as cytokine production, macrophage and synovial fibroblast responses, inflammasome activation, oxidative stress, and matrix degradation (17). *In vivo* studies could further test whether post-traumatic, obesity-related, and aging-related OA produce distinct inflammatory and tissue-remodeling trajectories (73).

Therapeutic development may benefit from strategies that address both mechanical drivers and inflammatory consequences. Weight management, exercise, rehabilitation, orthoses, and alignment correction may reduce upstream mechanical stress (3, 74, 75), whereas anti-inflammatory, metabolic, and mechanosensitive-pathway interventions may modify downstream biological responses (33). Whether patients with predominant mechanical abnormalities benefit from different strategies than those with synovitis, metabolic inflammation, or pain sensitization remains to be tested. This provides a rationale for evaluating stratified or combined approaches rather than assuming a uniform response to single-pathway interventions (76, 77). The central translational question is whether reproducible mechanical-inflammatory profiles can be measured prospectively and shown to predict treatment response.

### 4.5 Interdisciplinary integration and research priorities for the next decade

The journal-level and dual-map analyses showed that OA mechanoinflammation spans orthopedics, rheumatology, molecular and cell biology, biomechanics, biomaterials, and translational medicine. This interdisciplinary distribution reflects the need to consider mechanical exposure, stromal-cell signaling, innate inflammatory responses, tissue remodeling, pain, and clinical phenotype within a shared framework (3, 9, 78). However, the dispersed institutional and author-level collaboration networks suggest that methodological and organizational integration remains limited.

Future translational studies may therefore benefit from more integrated research designs. Clinical cohorts should collect mechanical, inflammatory, imaging, and symptom-related data in parallel so that these domains can be analyzed within the same framework (36). Longitudinal designs are especially important for determining whether mechanical-inflammatory and immune-related signatures predict structural progression, pain trajectories, or treatment response (68). Experimental studies should distinguish physiological from pathological loading and determine how inflammatory priming, tissue aging, and metabolic stress modify cellular responses. Multi-omics, imaging biomarkers, network analysis, and machine learning may help identify patient subgroups, but resulting models should be anchored in biologically plausible mechanical-inflammatory mechanisms rather than predictive associations alone (79). Greater standardization of loading protocols, inflammatory readouts, cell and tissue sources, and reporting practices will be necessary to improve comparability and clinical translation.

Over the next decade, the field is likely to advance through studies that connect mechanical and inflammatory information across scales rather than by expanding lists of isolated candidate mechanisms. Longitudinal cohorts that acquire biomechanical, imaging, inflammatory, molecular, symptom, and outcome data in parallel will be needed to test the stability and prognostic value of proposed endotypes (67, 68). In parallel, tissue-resolved molecular profiling and human-relevant experimental systems should clarify how local mechanical environments interact with cellular states across joint compartments (72, 79). These developments will be most informative when linked to biomarker-enriched intervention studies that test whether mechanistically defined subgroups predict treatment response. The central challenge is therefore to move from descriptive association toward prospective validation of mechanisms, models, and clinically useful stratification strategies.

### 4.6 Strengths and limitations

This study has several strengths. It addressed a clearly defined interdisciplinary field at the intersection of OA, mechanobiology, and inflammation. It combined WoSCC and PubMed to improve biomedical coverage while retaining WoSCC as the primary source for citation-based analyses. It integrated publication and collaboration analyses with reference co-citation, keyword co-occurrence, validated keyword–reference correspondence, citation-opportunity-adjusted temporal analysis, and journal-level knowledge-flow analyses. Threshold-, source-, retrieval-dependency-, and clustering-parameter sensitivity analyses further characterized the robustness and boundary dependence of the principal network structures. The literature-informed framework further integrates these findings across mechanical exposure, mechanosensing, inflammatory responses, tissue remodeling, and future research priorities.

Several limitations should also be acknowledged. First, database coverage, indexing differences, the English-language restriction, and metadata completeness may have influenced record inclusion despite the use of both WoSCC and PubMed. Second, citation-based indicators are influenced by publication age, journal visibility, disciplinary citation practices, and citation-accumulation time. Citation counts, co-citation strength, and total link strength therefore should not be interpreted as direct measures of scientific quality, biological importance, or therapeutic relevance. This limitation is particularly important for publications from the incomplete 2026 retrieval year. The opportunity-adjusted temporal analysis reduces the direct effect of unequal reference availability but cannot fully account for citation latency, visibility, or changes in the composition of the citing literature. Annual estimates, particularly for sparse early years and the incomplete 2026 year, should therefore be interpreted cautiously. Third, network structures depend on threshold selection, metadata standardization, keyword cleaning, and clustering algorithms. Sensitivity analyses confirmed that the exact number and boundaries of clusters vary with the VOSviewer resolution parameter, even when the main intermediate-resolution structures remain interpretable. Cluster labels should therefore be treated as analytical representations of thematic organization rather than fixed natural partitions. Fourth, bibliometric analysis identifies literature-level prominence and thematic associations but cannot establish biological causality, immune-cell function, therapeutic efficacy, or clinical utility. Accordingly, the prominence of an inflammatory pathway, immune-related term, or mechanosensitive target in the bibliometric network does not demonstrate its causal importance or therapeutic validity, and the proposed framework should be regarded as a hypothesis-generating synthesis. The proposed phenotyping, biomarker, stratification, and intervention elements are therefore research priorities rather than validated clinical tools. Several candidate mechanical features are primarily knee-OA examples and should not be generalized to other anatomical OA contexts without joint-specific validation.

## 5 Conclusion

This bibliometric and knowledge-map analysis shows that OA mechanoinflammation has expanded rapidly and developed into an increasingly interdisciplinary research area. At the primary analytical resolution, the literature is organized around complementary themes involving mechanical loading and mechanotransduction, inflammatory and matrix-remodeling responses, contemporary whole-joint pathogenesis and tissue crosstalk, and mechanosensitive-channel signaling. Validated keyword–reference correspondence and citation-opportunity-adjusted temporal analyses further indicate selective mechanotransduction-related relationships and greater recent relative use of contemporary OA and mechanosensitive-channel knowledge bases. These patterns support future investigation of multimodal phenotyping, biomarker validation, mechanically relevant models, and combined mechanical-inflammatory treatment strategies, while their clinical value remains to be established prospectively.

## Data Availability

The original contributions presented in the study are included in the article/**Supplementary Material**. Further inquiries can be directed to the corresponding authors.
