# Supplementary material for: Mechanoinflammation in osteoarthritis: knowledge evolution, cellular interactions, and translational research priorities
Source: Front Immunol. 2026 Oct 9;17:1955650. doi: 10.3389/fimmu.2026.1955650 (PMC13690958; doi:10.3389/fimmu.2026.1955650)
Supplement: Supplementary file 1 [file Table1.docx]

# Supplementary Tables

## **Supplementary Table S1. Parameters and thresholds for VOSviewer analyses.**

| **Analysis** | **Unit of analysis** | **Counting method** | **Main threshold** | **Clustering resolution** | **Minimum cluster size** | **Merge small clusters** |
| --- | --- | --- | --- | --- | --- | --- |
| Country/region collaboration | Countries/regions | Full counting | ≥3 publications | Not used for cluster interpretation | — | — |
| Institution collaboration | Institutions | Full counting | ≥3 publications | Not used for cluster interpretation | — | — |
| Author collaboration | Authors | Full counting | ≥3 publications | Not used for cluster interpretation | — | — |
| Reference co-citation | Cited references | Full counting | ≥10 citations | 1 | 3 | Yes |
| Author keyword co-occurrence | Author keywords | Full counting | ≥5 occurrences | 1 | 3 | Yes |
| Journal bibliographic coupling | Sources | Full counting | ≥3 documents | Not used for thematic interpretation | — | — |
| Cited-source co-citation | Cited sources | Full counting | ≥20 citations | 1 | 3 | Yes |

**Note**: VOSviewer analyses were performed using full counting. Clustering parameters are reported only for analyses in which cluster membership was substantively interpreted. Country/region, institution, and author collaboration maps were used to characterize collaboration connectivity and temporal publication patterns rather than cluster structure; therefore, collaboration-cluster counts were not interpreted or reported. For the principal thematic analyses, the clustering resolution was 1.00, the minimum cluster size was 3, and small-cluster merging was enabled. Threshold sensitivity analyses are reported in Supplementary Table S2, source-sensitivity analyses in Supplementary Table S3, and clustering-parameter sensitivity analyses in Supplementary Table S7.

## **Supplementary Table S2. Threshold sensitivity analysis of keyword and co-citation networks in the main dual-database dataset.**

| **Analysis** | **Main threshold** | **Tested threshold** | **Items** | **Clusters** | **Links** | **Total link strength** | **Interpretation** |
| --- | --- | --- | --- | --- | --- | --- | --- |
| Author keyword co-occurrence | 5 | 4 | 95 | 4 | 849 | 1911 | Four-cluster structure preserved with broader term inclusion |
| Author keyword co-occurrence | 5 | 5 | 66 | 4 | 560 | 1510 | Main analysis |
| Author keyword co-occurrence | 5 | 6 | 55 | 3 | 461 | 1357 | Boundary keyword themes merged under stricter threshold |
| Reference co-citation | 10 | 8 | 316 | 4 | 16395 | 25731 | Four-cluster intellectual structure preserved |
| Reference co-citation | 10 | 10 | 179 | 4 | 7122 | 13015 | Main analysis |
| Reference co-citation | 10 | 12 | 125 | 4 | 4136 | 8398 | Four-cluster intellectual structure preserved |
| Cited-source co-citation | 20 | 15 | 406 | 4 | 66698 | 1455789 | Four-cluster source-level structure preserved |
| Cited-source co-citation | 20 | 20 | 334 | 4 | 48464 | 1342740 | Main analysis |
| Cited-source co-citation | 20 | 25 | 281 | 4 | 35913 | 1242784 | Four-cluster source-level structure preserved |

**Note**: All analyses were performed using the main dual-database dataset (n = 554). The main thresholds were five occurrences for author keyword co-occurrence, ten citations for reference co-citation, and twenty citations for cited-source co-citation. Sensitivity analyses were performed by applying lower and higher thresholds while keeping the counting method, clustering resolution, minimum cluster size, and small-cluster merging settings consistent with the main analysis.

## **Supplementary Table S3. Source-sensitivity comparison between the dual-database and WoSCC-only datasets**

| **Analysis** | **Dual-database dataset** | **WoSCC-only dataset** | **Stability assessment** |
| --- | --- | --- | --- |
| Publications | 554 | 542 | 542/554 (97.8%) retained |
| PubMed-only publications | 12 | 0 | 2.2% of primary corpus |
| Author keyword items | 66 | 60 | Six boundary keywords absent in WoSCC-only analysis |
| Author-keyword clusters | 4 | 3 | Smaller boundary domains merged in WoSCC-only analysis |
| Cited sources | 334 | 332 | 332/334 (99.4%) retained |
| Cited-source clusters | 4 | 4 | Stable |
| Shared cited-source cluster assignments | 332/332 | — | 100% identical |
| Cited references | 179 | 177 | 177/179 (98.9%) retained |
| Reference co-citation clusters | 4 | 4 | Four-cluster solution retained |
| Shared reference cluster assignments | 174/177 | — | 98.3% identical |
| Reference co-citation ARI | — | 0.945 | High agreement |
| Reference co-citation NMI | — | 0.941 | High agreement |

**Note**: The WoSCC-only sensitivity analysis excluded the 12 PubMed-only records and their OpenAlex-linked cited-reference metadata. Reference co-citation analyses used the same threshold (≥10 citations), full-counting method, preprocessing procedures, and clustering settings as the primary analysis. Two references included in the primary network no longer met the citation threshold in the WoSCC-only dataset; both were threshold-boundary items. ARI, adjusted Rand index; NMI, normalized mutual information.

## **Supplementary Table S4. Post hoc corpus-coverage audit of mechanobiological concepts not explicitly enumerated in the retrieval query**

| **Mechanobiological concept** | **Records, n** | **Corpus, %** | **Title hits, n** | **Abstract hits, n** | **Author keyword hits, n** |
| --- | --- | --- | --- | --- | --- |
| Compression/compressive loading | 65 | 11.73 | 21 | 62 | 11 |
| Shear stress/strain | 22 | 3.97 | 9 | 22 | 8 |
| Integrin-related mechanisms | 18 | 3.25 | 5 | 18 | 6 |
| Primary cilia/cilium | 14 | 2.53 | 4 | 13 | 8 |
| Joint instability | 9 | 1.62 | 2 | 8 | 2 |
| Hydrostatic pressure | 8 | 1.44 | 4 | 7 | 2 |
| Matrix/substrate stiffness | 6 | 1.08 | 2 | 6 | 2 |
| Focal adhesion | 3 | 0.54 | 1 | 3 | 1 |

Note: Counts were determined from the titles, abstracts, and author keywords of the 554 publications included in the primary corpus. Because individual publications could contain more than one mechanobiological concept, counts across rows are not mutually exclusive and should not be summed. Overall, 127 of 554 publications (22.9%) contained at least one of the concepts listed above. This post hoc audit was conducted to assess whether mechanobiological concepts not explicitly enumerated as individual retrieval terms were nevertheless represented within the original corpus. It was used solely as a coverage assessment and did not modify the eligibility criteria or composition of the primary corpus. Keywords Plus were not included in the primary counts shown in this table.

## **Supplementary Table S5. Post hoc classification of retrieval dependence on channel-specific mechanosensing terms**

| **Retrieval category** | **All included records, n (%)** | **WoSCC records, n (%)** | **PubMed-only records, n (%)** |
| --- | --- | --- | --- |
| Generic mechanobiology terms only | 484 (87.36) | 474 (87.45) | 10 (83.33) |
| Generic + channel-specific terms | 46 (8.30) | 45 (8.30) | 1 (8.33) |
| Channel-specific terms only | 23 (4.15) | 23 (4.24) | 0 (0.00) |
| No reconstructed mechanobiology match | 1 (0.18) | 0 (0.00) | 1 (8.33) |
| Total | 554 (100.00) | 542 (100.00) | 12 (100.00) |

Note: Generic mechanobiology terms corresponded to the broader process-level descriptors specified in the original retrieval strategy. Channel-specific terms comprised PIEZO1, PIEZO2, Piezo channel(s), TRPV4, and transient receptor potential vanilloid 4. “Channel-specific terms only” indicates records that matched at least one channel-specific term but none of the generic mechanobiology terms within the database-specific retrieval fields. Percentages were calculated within each dataset. The single PubMed-only record classified as having no reconstructed mechanobiology match was retained because this post hoc classification was based on bibliographic metadata available after database harmonization and was not used to redefine the primary corpus.

## **Supplementary Table S6. Reproducibility audit of OpenAlex-assisted harmonization for PubMed-only records**

| **Year** | **PMID** | **DOI** | **OpenAlex Work ID** | **Cited references, n** | **Matching and validation status** |
| --- | --- | --- | --- | --- | --- |
| 2008 | 18464888 | 10.18926/amo/30956 | W1537788244 | 25 | Unique DOI match; PMID/title/year concordant |
| 2017 | 28955780 | 10.1016/j.bbrep.2017.06.006 | W2730425140 | 45 | Unique DOI match; PMID/title/year concordant |
| 2023 | 37662374 | 10.1101/2023.06.09.544419 | W4380265011 | 72 | Unique DOI match; PMID/title/year concordant |
| 2024 | 38692123 | 10.1016/j.plrev.2024.04.008 | W4394997547 | 36 | Unique DOI match; PMID/title/year concordant |
| 2025 | 40166233 | 10.1101/2025.03.17.643683 | W4408607241 | 38 | Unique DOI match; PMID/title/year concordant |
| 2025 | 40786277 | 10.7759/cureus.87711 | W4412193004 | 29 | Unique DOI match; PMID/title/year concordant |
| 2026 | 41757118 | 10.64898/2026.02.16.706240 | W7130343460 | 49 | Unique DOI match; PMID/title/year concordant |
| 2026 | 41865972 | 10.1016/j.joca.2026.03.121 | W7139918773 | 38 | Unique DOI match; PMID/title/year concordant |
| 2026 | 42107583 | 10.1016/j.joca.2026.04.018 | W7160663280 | 29 | Unique DOI match; PMID/title/year concordant |
| 2026 | 42119318 | 10.1016/j.compbiomed.2026.111737 | W7160919855 | 61 | Unique DOI match; PMID/title/year concordant |
| 2026 | 42127103 | 10.1073/pnas.2535302123 | W7161053703 | 95 | Unique DOI match; PMID/title/year concordant |
| 2026 | 42153977 | 10.1002/advs.75725 | W7161638074 | 44 | Unique DOI match; PMID/title/year concordant |

Note: Following cross-database deduplication, 12 of the 554 included publications were PubMed-only records requiring OpenAlex-assisted bibliographic harmonization. OpenAlex works were linked using exact DOI matching. All 12 records were uniquely matched, and PMID, normalized title, and publication year were concordant with the corresponding records in the original PubMed export. No ambiguous or unmatched records remained. OpenAlex metadata were accessed on May 24, 2026. Cited-reference counts represent references recovered from OpenAlex referenced_works. OpenAlex citation counts were not used as substitutes for WoSCC citation counts.

## **Supplementary Table S7. Sensitivity of principal cluster structures to VOSviewer clustering parameters**

| **Analysis** | **Resolution** | **Merge small clusters** | **Items** | **Clusters** | **ARI vs primary** | **NMI vs primary** | **Structural interpretation** |
| --- | --- | --- | --- | --- | --- | --- | --- |
| Author keyword co-occurrence | 1.00 | Yes | 66 | 4 | 1.000 | 1.000 | Primary configuration |
| Author keyword co-occurrence | 0.75 | Yes | 66 | 2 | 0.190 | 0.351 | K1-K3 largely coalesced into one broader domain; K4 remained distinct |
| Author keyword co-occurrence | 1.25 | Yes | 66 | 6 | 0.360 | 0.510 | Higher resolution subdivided mainly K1 and K2; K3 largely retained and K4 fully retained |
| Author keyword co-occurrence | 1.00 | No | 66 | 4 | 1.000 | 1.000 | Identical to primary configuration |
| Reference co-citation | 1.00 | Yes | 179 | 4 | 1.000 | 1.000 | Primary configuration |
| Reference co-citation | 0.75 | Yes | 179 | 2 | 0.463 | 0.501 | C1 and C3 largely coalesced; C2 and C4 largely coalesced |
| Reference co-citation | 1.25 | Yes | 179 | 6 | 0.661 | 0.722 | C3 and C4 highly coherent; C2 showed greatest subdivision and C1 limited subdivision |
| Reference co-citation | 1.00 | No | 179 | 4 | 1.000 | 1.000 | Identical to primary configuration |

Note: The primary clustering configuration used a resolution of 1.00, a minimum cluster size of 3, and small-cluster merging enabled. Sensitivity analyses altered one clustering parameter at a time while keeping the item-selection threshold, preprocessing, thesaurus, and counting method unchanged. Cluster labels are arbitrary across VOSviewer runs; structural interpretations were therefore based on item-level correspondence with the primary configuration rather than numerical cluster labels. ARI, adjusted Rand index; NMI, normalized mutual information.

## **Supplementary Table S8. Sensitivity of principal network structures to exclusion of records uniquely dependent on channel-specific retrieval terms**

Panel A. Author keyword co-occurrence (primary dual-database corpus vs channel-term-independent subset)

| **Metric** | **Primary 554** | **Channel-independent 531** | **Stability** |
| --- | --- | --- | --- |
| Source publications | 554 | 531 | 23 channel-specific-only records excluded |
| Keyword nodes | 66 | 64 | 64/66 (97.0%) retained |
| Clusters | 4 | 4 | Four-cluster solution retained |
| Links | 560 | 526 | 94.0% of primary links |
| Total link strength | 1,510 | 1,434 | 95.0% of primary TLS |
| Shared keyword nodes | — | 64 | — |
| Shared nodes retaining cluster assignment | — | 62/64 (96.9%) | High item-level agreement |
| Adjusted Rand index | — | 0.930 | High agreement |
| Normalized mutual information | — | 0.914 | High agreement |

Panel B. Reference co-citation (native WoSCC corpus vs channel-term-independent WoSCC subset)

| **Metric** | **WoSCC-only 542** | **Channel-independent 519** | **Stability** |
| --- | --- | --- | --- |
| Source publications | 542 | 519 | 23 channel-specific-only WoSCC records excluded |
| Cited references meeting threshold | 177 | 167 | 167/177 (94.4%) retained |
| Reference co-citation clusters | 4 | 4 | Four-cluster solution retained |
| Shared cited references | — | 167 | — |
| Shared references retaining cluster assignment | — | 164/167 (98.2%) | High item-level agreement |
| Adjusted Rand index | — | 0.952 | High agreement |
| Normalized mutual information | — | 0.935 | High agreement |
| Original C4 references retained above threshold | 24 | 23/24 | 95.8% retained |
| Retained original C4 references remaining in C4 | 24 | 23/23 (100%) | C4 membership preserved |
| Number of C4 references in resulting network | 24 | 24 | Domain retained |

Note: The channel-term-independent subsets excluded the 23 publications whose reconstructed mechanobiology retrieval match depended exclusively on PIEZO1/2- or TRPV4-specific terms and did not match any broader process-level mechanobiology descriptor. All sensitivity analyses used the same preprocessing, thresholds, counting method, thesaurus where applicable, and primary clustering settings as the corresponding main analyses. In the keyword network, TRPV4 and osteoclast fell below the ≥5-occurrence threshold after exclusion; among the 64 shared keyword nodes, only hyaluronic acid (K1→K4) and synovial fluid (K3→K4) changed cluster. In the reference network, ten references no longer met the ≥10-citation threshold; these were threshold-proximal items. ARI, adjusted Rand index; NMI, normalized mutual information.

## **Supplementary Table S9. Annual citation-opportunity-adjusted temporal analysis of reference co-citation clusters**

| **Year** | **Cluster** | **Citing docs, n** | **Available refs, n** | **Citation links, n** | **Citation opportunities** | **Rate per 1,000** | **Annual overall rate per 1,000** | **OAI** |
| --- | --- | --- | --- | --- | --- | --- | --- | --- |
| 2000 | C1 | 2 | 3 | 1 | 6 | 166.667 | 62.500 | 2.667 |
| 2000 | C2 | 2 | 13 | 1 | 26 | 38.462 | 62.500 | 0.615 |
| 2000 | C3 | 2 | 0 | 0 | 0 | NA | 62.500 | NA |
| 2000 | C4 | 2 | 0 | 0 | 0 | NA | 62.500 | NA |
| 2001 | C1 | 4 | 4 | 1 | 16 | 62.500 | 37.500 | 1.667 |
| 2001 | C2 | 4 | 16 | 2 | 64 | 31.250 | 37.500 | 0.833 |
| 2001 | C3 | 4 | 0 | 0 | 0 | NA | 37.500 | NA |
| 2001 | C4 | 4 | 0 | 0 | 0 | NA | 37.500 | NA |
| 2002 | C1 | 5 | 5 | 2 | 25 | 80.000 | 69.565 | 1.150 |
| 2002 | C2 | 5 | 18 | 6 | 90 | 66.667 | 69.565 | 0.958 |
| 2002 | C3 | 5 | 0 | 0 | 0 | NA | 69.565 | NA |
| 2002 | C4 | 5 | 0 | 0 | 0 | NA | 69.565 | NA |
| 2003 | C1 | 8 | 5 | 1 | 40 | 25.000 | 83.333 | 0.300 |
| 2003 | C2 | 8 | 22 | 17 | 176 | 96.591 | 83.333 | 1.159 |
| 2003 | C3 | 8 | 0 | 0 | 0 | NA | 83.333 | NA |
| 2003 | C4 | 8 | 0 | 0 | 0 | NA | 83.333 | NA |
| 2004 | C1 | 5 | 6 | 0 | 30 | 0.000 | 58.065 | 0.000 |
| 2004 | C2 | 5 | 25 | 9 | 125 | 72.000 | 58.065 | 1.240 |
| 2004 | C3 | 5 | 0 | 0 | 0 | NA | 58.065 | NA |
| 2004 | C4 | 5 | 0 | 0 | 0 | NA | 58.065 | NA |
| 2005 | C1 | 4 | 11 | 0 | 44 | 0.000 | 78.947 | 0.000 |
| 2005 | C2 | 4 | 27 | 12 | 108 | 111.111 | 78.947 | 1.407 |
| 2005 | C3 | 4 | 0 | 0 | 0 | NA | 78.947 | NA |
| 2005 | C4 | 4 | 0 | 0 | 0 | NA | 78.947 | NA |
| 2006 | C1 | 7 | 14 | 4 | 98 | 40.816 | 74.830 | 0.545 |
| 2006 | C2 | 7 | 28 | 18 | 196 | 91.837 | 74.830 | 1.227 |
| 2006 | C3 | 7 | 0 | 0 | 0 | NA | 74.830 | NA |
| 2006 | C4 | 7 | 0 | 0 | 0 | NA | 74.830 | NA |
| 2007 | C1 | 9 | 18 | 5 | 162 | 30.864 | 56.738 | 0.544 |
| 2007 | C2 | 9 | 29 | 19 | 261 | 72.797 | 56.738 | 1.283 |
| 2007 | C3 | 9 | 0 | 0 | 0 | NA | 56.738 | NA |
| 2007 | C4 | 9 | 0 | 0 | 0 | NA | 56.738 | NA |
| 2008 | C1 | 8 | 19 | 14 | 152 | 92.105 | 85.000 | 1.084 |
| 2008 | C2 | 8 | 31 | 20 | 248 | 80.645 | 85.000 | 0.949 |
| 2008 | C3 | 8 | 0 | 0 | 0 | NA | 85.000 | NA |
| 2008 | C4 | 8 | 0 | 0 | 0 | NA | 85.000 | NA |
| 2009 | C1 | 8 | 21 | 2 | 168 | 11.905 | 25.463 | 0.468 |
| 2009 | C2 | 8 | 32 | 9 | 256 | 35.156 | 25.463 | 1.381 |
| 2009 | C3 | 8 | 0 | 0 | 0 | NA | 25.463 | NA |
| 2009 | C4 | 8 | 1 | 0 | 8 | 0.000 | 25.463 | 0.000 |
| 2010 | C1 | 9 | 29 | 6 | 261 | 22.989 | 27.350 | 0.841 |
| 2010 | C2 | 9 | 33 | 10 | 297 | 33.670 | 27.350 | 1.231 |
| 2010 | C3 | 9 | 1 | 0 | 9 | 0.000 | 27.350 | 0.000 |
| 2010 | C4 | 9 | 2 | 0 | 18 | 0.000 | 27.350 | 0.000 |
| 2011 | C1 | 7 | 33 | 10 | 231 | 43.290 | 41.096 | 1.053 |
| 2011 | C2 | 7 | 36 | 11 | 252 | 43.651 | 41.096 | 1.062 |
| 2011 | C3 | 7 | 1 | 0 | 7 | 0.000 | 41.096 | 0.000 |
| 2011 | C4 | 7 | 3 | 0 | 21 | 0.000 | 41.096 | 0.000 |
| 2012 | C1 | 9 | 40 | 13 | 360 | 36.111 | 32.922 | 1.097 |
| 2012 | C2 | 9 | 37 | 11 | 333 | 33.033 | 32.922 | 1.003 |
| 2012 | C3 | 9 | 1 | 0 | 9 | 0.000 | 32.922 | 0.000 |
| 2012 | C4 | 9 | 3 | 0 | 27 | 0.000 | 32.922 | 0.000 |
| 2013 | C1 | 11 | 49 | 25 | 539 | 46.382 | 49.407 | 0.939 |
| 2013 | C2 | 11 | 39 | 25 | 429 | 58.275 | 49.407 | 1.179 |
| 2013 | C3 | 11 | 1 | 0 | 11 | 0.000 | 49.407 | 0.000 |
| 2013 | C4 | 11 | 3 | 0 | 33 | 0.000 | 49.407 | 0.000 |
| 2014 | C1 | 12 | 51 | 17 | 612 | 27.778 | 43.729 | 0.635 |
| 2014 | C2 | 12 | 44 | 35 | 528 | 66.288 | 43.729 | 1.516 |
| 2014 | C3 | 12 | 1 | 0 | 12 | 0.000 | 43.729 | 0.000 |
| 2014 | C4 | 12 | 5 | 1 | 60 | 16.667 | 43.729 | 0.381 |
| 2015 | C1 | 11 | 54 | 18 | 594 | 30.303 | 24.871 | 1.218 |
| 2015 | C2 | 11 | 44 | 10 | 484 | 20.661 | 24.871 | 0.831 |
| 2015 | C3 | 11 | 2 | 0 | 22 | 0.000 | 24.871 | 0.000 |
| 2015 | C4 | 11 | 6 | 1 | 66 | 15.152 | 24.871 | 0.609 |
| 2016 | C1 | 17 | 57 | 41 | 969 | 42.312 | 24.987 | 1.693 |
| 2016 | C2 | 17 | 45 | 5 | 765 | 6.536 | 24.987 | 0.262 |
| 2016 | C3 | 17 | 5 | 1 | 85 | 11.765 | 24.987 | 0.471 |
| 2016 | C4 | 17 | 6 | 1 | 102 | 9.804 | 24.987 | 0.392 |
| 2017 | C1 | 22 | 62 | 41 | 1364 | 30.059 | 23.094 | 1.302 |
| 2017 | C2 | 22 | 46 | 16 | 1012 | 15.810 | 23.094 | 0.685 |
| 2017 | C3 | 22 | 8 | 3 | 176 | 17.045 | 23.094 | 0.738 |
| 2017 | C4 | 22 | 8 | 3 | 176 | 17.045 | 23.094 | 0.738 |
| 2018 | C1 | 14 | 63 | 23 | 882 | 26.077 | 27.559 | 0.946 |
| 2018 | C2 | 14 | 48 | 18 | 672 | 26.786 | 27.559 | 0.972 |
| 2018 | C3 | 14 | 8 | 3 | 112 | 26.786 | 27.559 | 0.972 |
| 2018 | C4 | 14 | 8 | 5 | 112 | 44.643 | 27.559 | 1.620 |
| 2019 | C1 | 28 | 64 | 100 | 1792 | 55.804 | 35.714 | 1.562 |
| 2019 | C2 | 28 | 50 | 22 | 1400 | 15.714 | 35.714 | 0.440 |
| 2019 | C3 | 28 | 13 | 5 | 364 | 13.736 | 35.714 | 0.385 |
| 2019 | C4 | 28 | 9 | 9 | 252 | 35.714 | 35.714 | 1.000 |
| 2020 | C1 | 27 | 66 | 87 | 1782 | 48.822 | 30.531 | 1.599 |
| 2020 | C2 | 27 | 52 | 20 | 1404 | 14.245 | 30.531 | 0.467 |
| 2020 | C3 | 27 | 18 | 8 | 486 | 16.461 | 30.531 | 0.539 |
| 2020 | C4 | 27 | 12 | 7 | 324 | 21.605 | 30.531 | 0.708 |
| 2021 | C1 | 37 | 66 | 97 | 2442 | 39.722 | 37.871 | 1.049 |
| 2021 | C2 | 37 | 54 | 84 | 1998 | 42.042 | 37.871 | 1.110 |
| 2021 | C3 | 37 | 27 | 24 | 999 | 24.024 | 37.871 | 0.634 |
| 2021 | C4 | 37 | 15 | 22 | 555 | 39.640 | 37.871 | 1.047 |
| 2022 | C1 | 42 | 66 | 130 | 2772 | 46.898 | 35.366 | 1.326 |
| 2022 | C2 | 42 | 54 | 50 | 2268 | 22.046 | 35.366 | 0.623 |
| 2022 | C3 | 42 | 33 | 56 | 1386 | 40.404 | 35.366 | 1.142 |
| 2022 | C4 | 42 | 18 | 18 | 756 | 23.810 | 35.366 | 0.673 |
| 2023 | C1 | 45 | 66 | 93 | 2970 | 31.313 | 31.692 | 0.988 |
| 2023 | C2 | 45 | 54 | 58 | 2430 | 23.868 | 31.692 | 0.753 |
| 2023 | C3 | 45 | 35 | 74 | 1575 | 46.984 | 31.692 | 1.483 |
| 2023 | C4 | 45 | 21 | 26 | 945 | 27.513 | 31.692 | 0.868 |
| 2024 | C1 | 49 | 66 | 104 | 3234 | 32.158 | 44.465 | 0.723 |
| 2024 | C2 | 49 | 54 | 99 | 2646 | 37.415 | 44.465 | 0.841 |
| 2024 | C3 | 49 | 35 | 103 | 1715 | 60.058 | 44.465 | 1.351 |
| 2024 | C4 | 49 | 24 | 84 | 1176 | 71.429 | 44.465 | 1.606 |
| 2025 | C1 | 85 | 66 | 187 | 5610 | 33.333 | 33.125 | 1.006 |
| 2025 | C2 | 85 | 54 | 67 | 4590 | 14.597 | 33.125 | 0.441 |
| 2025 | C3 | 85 | 35 | 139 | 2975 | 46.723 | 33.125 | 1.410 |
| 2025 | C4 | 85 | 24 | 111 | 2040 | 54.412 | 33.125 | 1.643 |
| 2026* | C1 | 57 | 66 | 83 | 3762 | 22.063 | 37.930 | 0.582 |
| 2026* | C2 | 57 | 54 | 83 | 3078 | 26.966 | 37.930 | 0.711 |
| 2026* | C3 | 57 | 35 | 109 | 1995 | 54.637 | 37.930 | 1.440 |
| 2026* | C4 | 57 | 24 | 112 | 1368 | 81.871 | 37.930 | 2.158 |

Note: The temporal analysis used the 542 native WoSCC citing publications and retained the fixed 179 cited references and their original C1–C4 assignments from the primary co-citation network. Citing docs, n is the number of native WoSCC corpus publications in year y; Available refs, n is the number of fixed cluster references published by year y and therefore available for citation; Citation links, n is the number of links from year-y citing publications to those available cluster references; Citation opportunities = Citing docs × Available refs; Rate per 1,000 = 1,000 × Citation links / Citation opportunities; Annual overall rate is calculated across all C1–C4 references available in the same year; OAI = cluster-specific rate / annual overall rate. NA indicates that no reference in the corresponding cluster was yet available for citation. *2026 was incomplete at the retrieval date.

## **Supplementary Table S10. Complete validated keyword–reference correspondence results for all 16 K–C comparisons**

| **Pair** | **Observed cosine** | **95% bootstrap CI** | **Null mean** | **Excess** | **Empirical P** | **FDR q** | **n(K)** | **n(C)** | **n(K∩C)** | **FDR significant** |
| --- | --- | --- | --- | --- | --- | --- | --- | --- | --- | --- |
| K1–C1 | 0.704 | 0.666–0.737 | 0.701 | 0.003 | 0.4458 | 0.8915 | 397 | 371 | 270 | No |
| K1–C3 | 0.594 | 0.551–0.635 | 0.558 | 0.035 | 0.0071 | 0.0284 | 397 | 214 | 173 | Yes |
| K1–C2 | 0.536 | 0.491–0.579 | 0.552 | -0.016 | 0.8661 | 0.9992 | 397 | 242 | 166 | No |
| K2–C2 | 0.524 | 0.465–0.577 | 0.379 | 0.145 | 0.0001 | 0.0008 | 166 | 242 | 105 | Yes |
| K1–C4 | 0.476 | 0.430–0.523 | 0.468 | 0.008 | 0.3593 | 0.8212 | 397 | 152 | 117 | No |
| K3–C2 | 0.416 | 0.353–0.472 | 0.340 | 0.076 | 0.0023 | 0.0123 | 131 | 242 | 74 | Yes |
| K2–C1 | 0.395 | 0.343–0.445 | 0.430 | -0.035 | 0.9722 | 0.9992 | 166 | 371 | 98 | No |
| K2–C4 | 0.390 | 0.322–0.455 | 0.277 | 0.114 | 0.0001 | 0.0008 | 166 | 152 | 62 | Yes |
| K3–C1 | 0.372 | 0.317–0.421 | 0.381 | -0.009 | 0.7225 | 0.9992 | 131 | 371 | 82 | No |
| K2–C3 | 0.361 | 0.296–0.421 | 0.329 | 0.032 | 0.0977 | 0.2605 | 166 | 214 | 68 | No |
| K3–C4 | 0.276 | 0.211–0.344 | 0.210 | 0.067 | 0.0109 | 0.0349 | 131 | 152 | 39 | Yes |
| K4–C1 | 0.264 | 0.206–0.315 | 0.287 | -0.024 | 0.8948 | 0.9992 | 75 | 371 | 44 | No |
| K3–C3 | 0.227 | 0.169–0.285 | 0.241 | -0.014 | 0.7734 | 0.9992 | 131 | 214 | 38 | No |
| K4–C3 | 0.182 | 0.121–0.242 | 0.182 | -0.000 | 0.5705 | 0.9992 | 75 | 214 | 23 | No |
| K4–C2 | 0.156 | 0.100–0.213 | 0.238 | -0.082 | 0.9992 | 0.9992 | 75 | 242 | 21 | No |
| K4–C4 | 0.131 | 0.072–0.192 | 0.163 | -0.032 | 0.8986 | 0.9992 | 75 | 152 | 14 | No |

Note: Observed cosine was calculated from binary document-incidence vectors for each author keyword cluster (K1–K4) and reference co-citation cluster (C1–C4). Null mean and empirical P values were obtained from 10,000 year-stratified permutations in which complete reference-cluster profiles were reassigned among publications within publication year while keyword profiles were held fixed. FDR q values were obtained using Benjamini–Hochberg correction across all 16 comparisons. The 95% CI was estimated using 2,000 year-stratified paired bootstrap resamples. n(K) and n(C) are the numbers of publications assigned to the respective keyword and reference clusters; n(K∩C) is the number assigned to both.
